# Deciphering heterogeneity of septic shock patients using immune functional assays: a proof of concept study

**DOI:** 10.1038/s41598-020-73014-2

**Published:** 2020-09-30

**Authors:** Chloé Albert Vega, Guy Oriol, François Bartolo, Jonathan Lopez, Alexandre Pachot, Thomas Rimmelé, Fabienne Venet, Véronique Leray, Guillaume Monneret, Benjamin Delwarde, Karen Brengel-Pesce, Julien Textoris, François Mallet, Sophie Trouillet-Assant

**Affiliations:** 1 Laboratoire Commun de Recherche Hospices Civils de Lyon-bioMérieux, Joint Research Unit Hospices Civils de Lyon-bioMérieux, Hospices Civils de Lyon, Centre Hospitalier Lyon Sud, Bâtiment 3F, Pierre-Bénite, 69495 Lyon, France; 2 Open Innovation and Partnerships Department, bioMérieux S.A., Marcy l’Étoile, France; 3 Soladis, Lyon, France; 4 Hospices Civils de Lyon - Plateforme de Recherche de Transfert en Oncologie, Centre Hospitalier Lyon Sud, Pierre-Bénite, 69495 Lyon, France; 5 EA 7426 Pathophysiology of Injury-Induced Immunosuppression, PI3, Claude Bernard Lyon 1 University-bioMérieux-Hospices Civils de Lyon, Hôpital Edouard Herriot, 69437 Lyon, France; 6 Anesthesia and Critical Care Medicine Department, Hôpital Edouard Herriot, Hospices Civils de Lyon, 69437 Lyon, France; 7 Immunology Laboratory, Hôpital Edouard Herriot, Hospices Civils de Lyon, 69437 Lyon, France; 8 CIRI, INSERM U1111, CNRS 5308, ENS, UCBL, Faculté de Médecine Lyon Est, Virpath - Université Lyon, 7 rue Guillaume Paradin, 69372 Lyon Cedex 08, France

**Keywords:** Immunology, Biomarkers

## Abstract

The complexity of sepsis pathophysiology hinders patient management and therapeutic decisions. In this proof-of-concept study we characterised the underlying host immune response alterations using a standardised immune functional assay (IFA) in order to stratify a sepsis population. In septic shock patients, ex vivo LPS and SEB stimulations modulated, respectively, 5.3% (1/19) and 57.1% (12/21) of the pathways modulated in healthy volunteers (HV), highlighting deeper alterations induced by LPS than by SEB. SEB-based clustering, identified 3 severity-based groups of septic patients significantly different regarding mHLA-DR expression and TNFα level post-LPS, as well as 28-day mortality, and nosocomial infections. Combining the results from two independent cohorts gathering 20 HV and 60 patients, 1 cluster grouped all HV with 12% of patients. The second cluster grouped 42% of patients and contained all non-survivors. The third cluster grouped 46% of patients, including 78% of those with nosocomial infections. The molecular features of these clusters indicated a distinctive contribution of previously described genes defining a “healthy-immune response” and a “sepsis-related host response”. The third cluster was characterised by potential immune recovery that underlines the possible added value of SEB-based IFA to capture the sepsis immune response and contribute to personalised management.

## Introduction

Sepsis has been acknowledged as a global health priority by the World Health Organization in 2017^1^, representing a worldwide health problem in terms of morbidity and mortality, as well as social and economic costs. Since 2016, the official definition of sepsis is a life-threatening organ dysfunction caused by a dysregulated host response to infection^2^. Increasing knowledge concerning sepsis has led to the realisation that septic patients’ immune reaction is a complex and ongoing process rather than a compartmentalized sequence of immune responses. Patients’ blood typically represents a mixed hyperinflammatory and immunosuppressing milieu that coexists and changes over the course of sepsis^3,4^. Hence, therapies directly acting on the immune system seem a promising approach for sepsis management^5^. Both phases have been targeted in sepsis patients, using either anti-inflammatory agents to counteract the hyper-inflammatory phase^6,7^, or immunotherapy to try to boost the immune system in order to reduce the immunocompromised phase in septic shock^8–10^ yet no benefit from either therapy has been found^11^. This may be due to the complexity of sepsis pathophysiology, including inter-individual variability of the immune response^12^ and the underlying immune defects of individual patients^13^. In addition, the host immune response evolves over the course of sepsis^14^ varying the intensity of the net immune balance^15^. Patient stratification according to immunological profiles could therefore be a key step towards successful care^16^. A diagnostic tool allowing the precise identification of the immune status and immune function at a given time would therefore be crucial to personalise treatment decisions.

Interpreting the host response of septic patients remains a challenge. For instance, soluble and cell surface markers, such as monocyte HLA-DR^17^ and neutrophil CD88 expression^18^, as well as lymphocyte or platelet count, remain a single cell population measure, which is likely to underestimate the overall immune contribution of all cell types involved. In other clinical situations where circulating biomarkers failed to diagnose infection (e.g. latent tuberculosis infection) or stratify patients, the use of immune functional assays (IFA) has considerably improved patient management^19–22^. These can directly document, ex vivo, the capacity of a cell population to respond to an immune challenge. They have also occasionally been used in the research setting to investigate the anergy of monocytes, most frequently by measuring TNFα protein after ex vivo lipopolysaccharide (LPS) stimulation^23–25^. We hypothesised that IFAs could allow an accurate description, at a given time point, of the overall immune response capacity of septic patients, independently of the previous pathogen exposure.

In this proof-of-concept study, we evaluated the potential of using a standardised IFA for the stratification of a septic population based on immune alterations. We developed an IFA using LPS, the reference stimulation agent for these assays in sepsis, and the superantigen staphylococcal enterotoxin B (SEB) as stimuli. LPS signals through the innate receptor TLR-4 and -2 which triggers the innate immunity^26,27^, while SEB provokes polyclonal T cell activation, bridging T cells to antigen presenting cells, among which dendritic cells and macrophages, triggering the innate immunity as well as initiating the adaptive response^28,29^. To evaluate the response to LPS or SEB, an 86-gene custom panel was designed, among which a 44-gene signature defining a “healthy immune response”, and 42 genes related to a “sepsis-related host response”, such as *ADGRE3*^30^, *CD74*^31^ and *CLEC7A*^32^. Immune alterations were identified in two prospective cohorts of septic patients according to the Sepsis-3 definition. The use of LPS allowed to detect a profound alteration of the innate immune arm, while heterogeneity in the response of the adaptive immune arm was revealed after SEB challenge. Importantly, the SEB-based IFA allowed the stratification of septic patients according to their immune profile in an unprecedented way.

## Results

### Clinical characteristics of patients and healthy volunteers

#### Discovery cohort

From June 2017 to June 2018, 30 patients with septic shock, according to the Sepsis-3 definition, were included; the median [IQR] age of patients was 66 [59–73] years, and 70% were male. Definition of shock included the use of vasopressor, the median [IQR] duration of which was 3.5 [2.0–6.8] days, and serum lactate > 2 mmol/L, the median [IQR] concentration of which was 3.2 [2.6–5.2] mmol/L. At day 1–2, the median [IQR] SOFA score was 8 [7–10] and SAPS II was 59 [48–77]. The majority of septic shock patients (90%) had low mHLA-DR (< 15,000 AB/c) 3–4 days after shock onset. During intensive care unit (ICU) stay, mortality at day 28 was 13.3% (n = 4) and the median ICU stay among those who were discharged alive was 7.5 days. Further details on the clinical and immunological characteristics of patients are described in Table 1, while the presence and nature of comorbidities are presented in Supplementary Table S1, and number of infections and pathogen responsible for sepsis are presented in Supplementary Table S2.

**Table 1:** Clinical and immunological data for patients with septic shock.

	Septic shock patients (n = 30)
**Admission data**	
Sex, male, n (%)	21 (70)
Median age, years [IQR]	66 [59–73]
Median BMI, kg/m2 [IQR]	27 [21–34]
Median SAPS II [IQR]	59 [48–77]
Median SOFA score (day 1) [IQR]	8 [7–10]
Median plasma lactate level, mM [IQR]	3.2 [2.6–5.2]
Median CCI [IQR]	1.5 [0.1–3.3]
Comorbidities^a^, n (%)	
0	10 (33.3)
≥ 1	20 (66.7)
Primary site of infection, n (%)	
Abdominal	9 (30)
UTI	6 (20)
SST	4 (13)
Others	11 (37)
Type of primary infection, n (%)	
Community acquired	13 (43)
Hospital acquired	17 (57)
Documentation of infection, n (%)	
Gram-negative	7 (23.3)
Gram-positive	6 (20)
Virus	1 (3.3)
Co-infection	6 (20)
Non-documented infection	10 (33.3)
Hydrocortisone, n (%)	10 (33)
**Day 3–4 data**	
Immunology	
Median mHLA-DR, Ab/C [IQR]	7348 [3838–10103]
Median TNFα secretion post-LPS stimulation, pg/mL [IQR]	701 [320–1260]
**Outcomes**	
Vasopressor requirement, n (%)	30 (100)
Median vasopressor duration, days [IQR]	3.5 [2–6.8]
Hemofiltration, n (%)	10 (33)
Mechanical ventilation, n (%)	22 (73)
Median ICU length of stay, days [IQR]	8 [4.2–12]
Median hospital length of stay, days [IQR]	56 [20–78]
Mortality at day 28, n (%)	4 (13.3)

SAPS II was calculated after admission and SOFA score was measured after 24 h of ICU stay.

*BMI* body mass index, *SAPS II* simplified acute physiology score, *SOFA* sequential organ failure assessment, *CCI* Charlson comorbidity index, *UTI* urinary tract infection, *SST* skin and soft tissue, *HLA-DR* human leukocyte antigen DR, *TNFα* tumour necrosis factor alpha, *LPS* lipopolysaccharide, *ICU* intensive care unit.

^a^Presence of comorbidities was affirmative when at least one of the following comorbidity was present in the patient: chronic pulmonary disease, heart failure, myocardial infarction, ulcer, diabetes, renal failure, or malign solid tumour.

Concomitantly, a total of 10 healthy volunteers (HV) were included; their median [IQR] age was 54 [51–57] years, and 50% were male.

### Modulation of immune response

**Figure 1:**
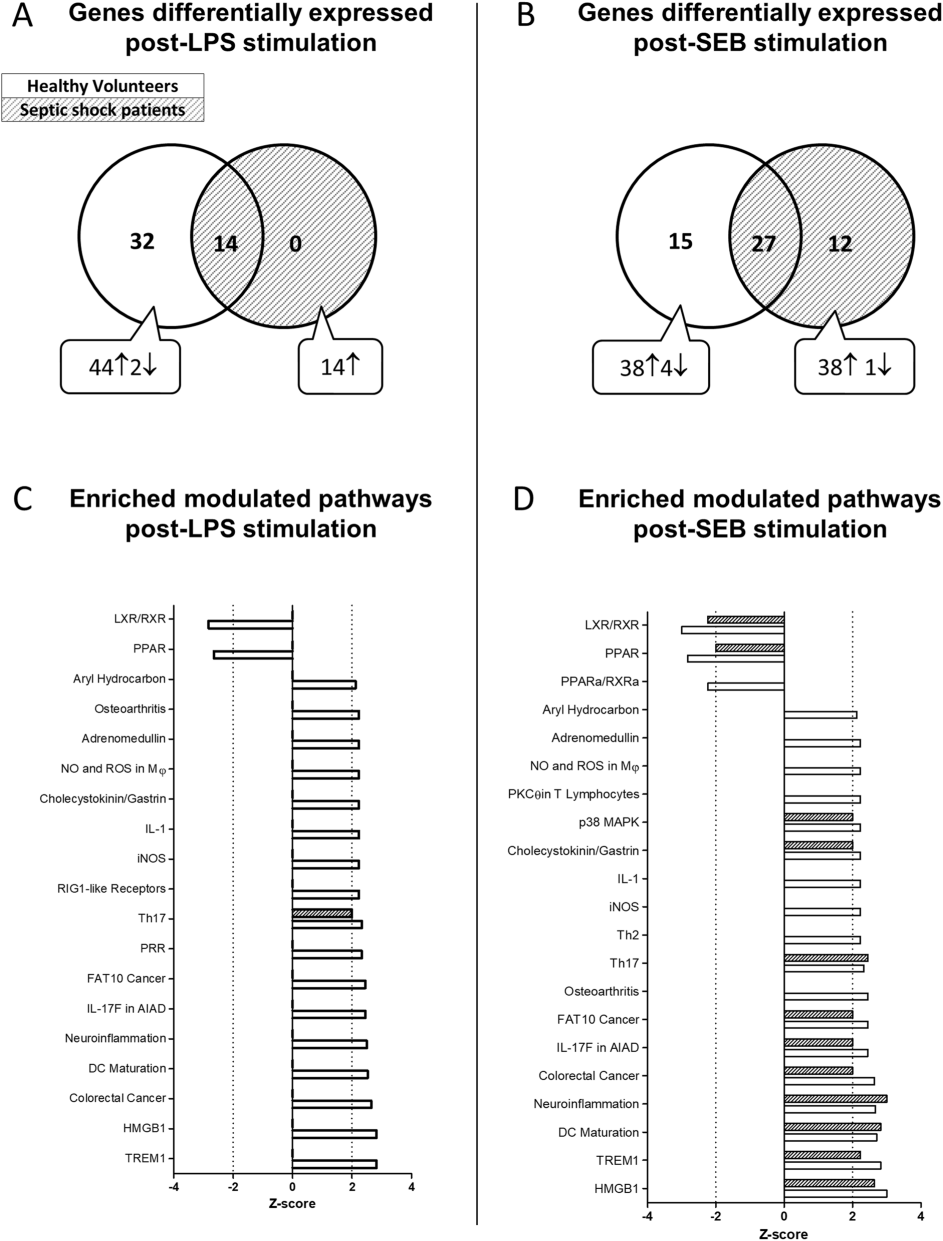
Contrasted response to LPS and SEB stimulation in healthy volunteers (HV) and septic shock patients. Venn diagrams for differentially expressed genes (adjusted *p* value < 0.05 in > 75% of tests) between 10 HV (white circle) and 30 septic shock patients (dashed circle) (**A**) upon LPS or (**B**) SEB stimulation for 24 h. Enriched-modulated pathways (Z-score positive activation, Z-score negative inhibition) derived from differentially expressed genes (using ingenuity analysis) for HV (solid bars) and septic shock patients (hashed bars) (**C**) upon LPS and (**D**) SEB stimulation. *LPS* lipopolysaccharide, *SEB* staphylococcal enterotoxin B.

Heparinised-whole blood from HV was incubated in TruCulture tubes pre-filled with LPS or SEB to describe the healthy immune response. The molecular immune response strongly correlated with the published results for both LPS (Supplementary Figure S1A) and SEB (Supplementary Figure S1B; Spearman rho > 0.9 for both, *p* < 0.001), ensuring the robustness of the experimental process and allowing the validation of the IFA used herein. Similarly, heparinised-whole blood from patients, collected at day 3–4 after septic shock onset, was used for immune response evaluation. In order to compare the response of HV to that of septic shock patients following an immune challenge, results of the stimulation condition (using either LPS or SEB) were normalised to those of the control condition (NUL; ratio stimulation condition/NUL condition). After LPS stimulation, the median [IQR] concentration of TNFα was significantly lower in septic shock patients (701 [320–1260] pg/mL) than in HV (5214 [4202–5709] pg/mL, *p* < 0.0001; supplementary Figure S2A), as was *TNFA* gene induction (fold change 1.4 [0.9–2.0] septic shock patients vs. 6.9 [4.9–7.8] HV, *p* < 0.0001; supplementary Figure S2B); these values were significantly correlated (Spearman rho = 0.75, *p* < 0.001, supplementary Figure S2C). Upon LPS stimulation, there were 46/86 (53.5%) differentially expressed genes in HV and 14/86 (16.3%) in septic shock patients; all genes for which expression was increased by LPS in septic shock patients were also increased in HV (Fig. 1A). Upon SEB stimulation, 42/86 (48.8%) genes in HV and 39/86 (45.3%) in septic shock patients were differentially expressed; 12/39 (31%) genes for which expression increased in response to SEB stimulation were specific to septic shock patients (*BST2*, *CD74*, *HLA-DPA1*, *HLA-DRA*, *IRF7*, *MX1*, *OAS1*, *OAS2*, *RARRES3*, *SLAMF7*, *STAT2*, *TNFSF13B*; Fig. 1B).

To better understand the underlying biological differences, we examined the pathways activated upon each stimulation for both HV and septic shock patients using ingenuity pathway analysis. In HV, LPS stimulation was responsible for the modulation of 19 canonical pathways while SEB stimulation modulated 21 canonical pathways (Fig. 1C, D). Pathways uniquely induced by LPS stimulation were RIG1-like receptors in innate immunity and the recognition of PAMPs through pattern recognition receptor (PRR pathway; Fig. 1C). Pathways specifically activated upon SEB stimulation were related to T cell activation and regulation signalling via PKCθ in T lymphocytes, p38 MAPK signalling, and Th2 pathway (Fig. 1D). Of note, no additional pathway was found to be modulated in septic shock patients compared to HV. LPS stimulation modulated 1/19 pathways in septic shock patients (Fig. 1C), whereas SEB stimulation modulated 12/21 pathways in septic shock patients (Fig. 1D).

### The miscellaneous response to LPS and SEB challenges

**Figure 2:**
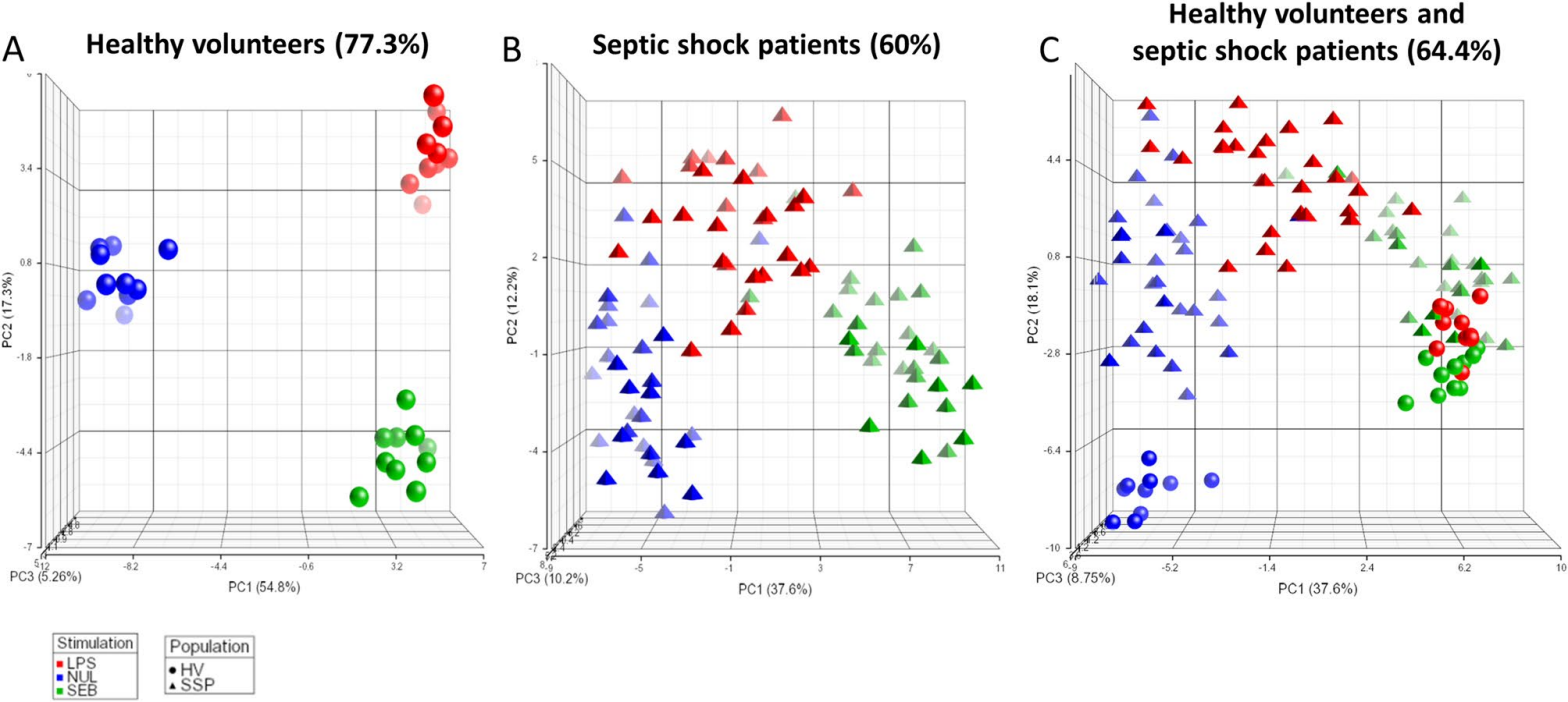
Spatial distribution of healthy volunteers (HV) and septic shock patient’s response according to LPS and SEB stimulation. PCA of immune responses for (**A**) 10 HV (circles), (**B**) 30 septic shock patients (triangles) and (**C**) both populations after LPS (red) or SEB (green) stimulation, as well as basal condition (NUL; blue). The percentage of variance captured by each PC axis is indicated, as well as the total variance. Vector position for each sample was plotted and visualisation was executed with Partek. *LPS* lipopolysaccharide, *SEB* staphylococcal enterotoxin B, *PCA* principal component analysis, *PC* principal component.

An unsupervised analysis of the data, using the full gene panel, showed a robust space aggregation for each experimental condition (i.e. NUL, LPS stimulation, and SEB stimulation) for HV (77.3%), whereas a more heterogeneous distribution and lower explained variance was observed in septic shock patients (60%; Fig. 2A-B). The molecular read-out captures the diversity of each stimulation and discriminates septic patients from a healthy population (Fig. 2C).

**Figure 3:**
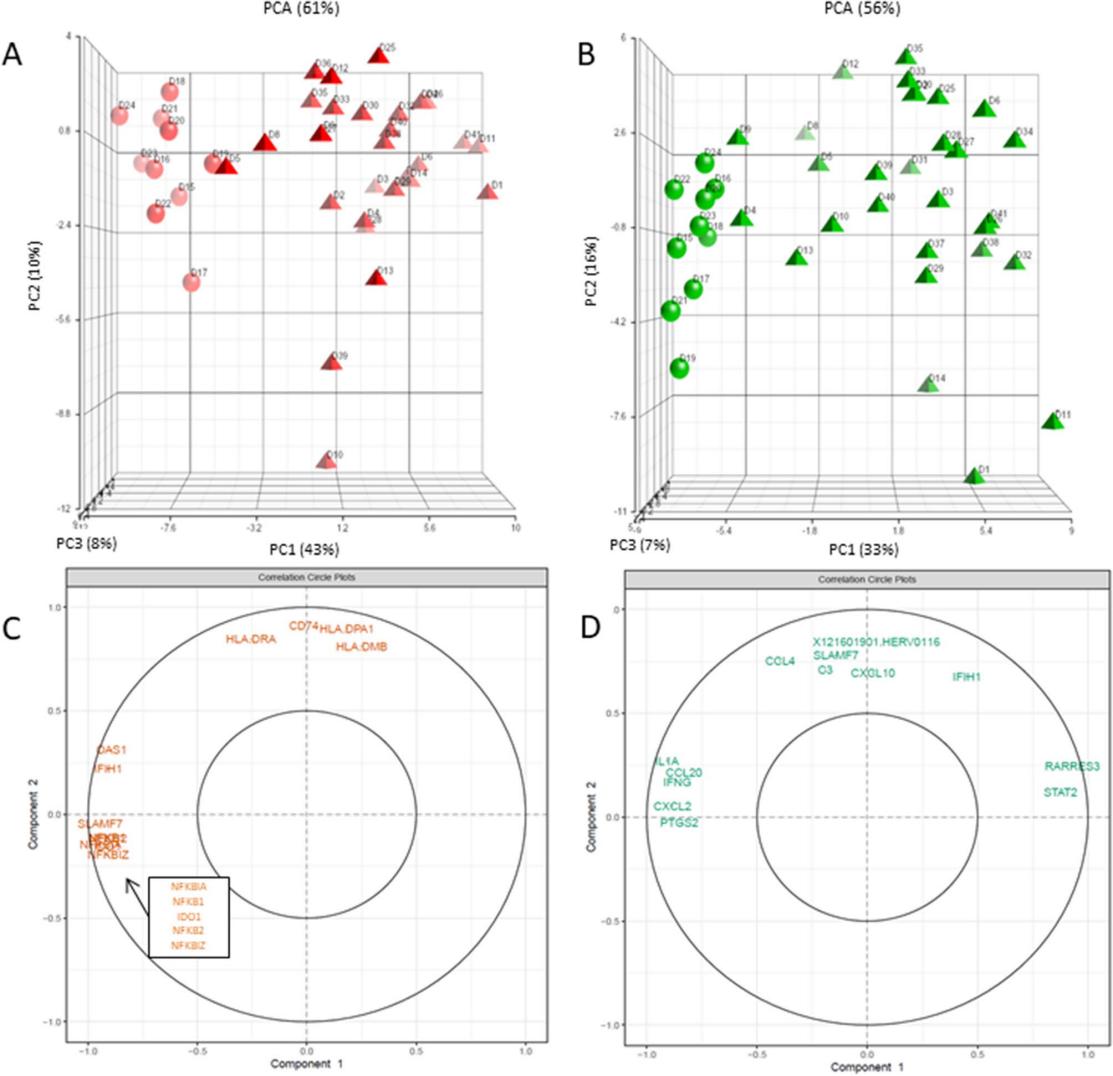
Genes contributing to the higher variance in responses following LPS and SEB stimulation for healthy volunteers (HV) and septic shock patients. PCA of 10 HV (circles) and 30 septic shock patients (triangles) response (stimulation/NUL) to (**A**) LPS stimulation (red) and (**B**) SEB stimulation (green). Every donor is labelled. The percentage of variance captured by each PC axis is indicated, as well as the total variance. Vector position for each donor were plotted and visualisation was executed with Partek. The most important variables are plotted (representing 15% of the total variables weight for PC1 and PC2) for (**C**) LPS response and (**D**) SEB response. *LPS* lipopolysaccharide, *SEB* staphylococcal enterotoxin B, *PCA* principal component analysis, *PC* principal component.

To discriminate which genes mainly contributed to quantitative variations for each stimulation, LPS (Fig. 3A) and SEB (Fig. 3B) responses for HV and septic shock patients were plotted and the weight of the genes explaining the variance was obtained (Supplementary Table S3 and S4). Among the greatest contributors to LPS response variance for the first component (43%) discriminating both populations, *SLAMF7*, *NFκB*-related genes (*NFκBIA, NFκB1*), *IDO1*, and *IFIH1* were found to be more greatly expressed by HV than by septic shock patients (Fig. 3C). Among the greatest contributors to SEB response variance for the first component (33%), *RARRES3* and *STAT2* were found to be more greatly expressed by individuals on the right side of the component, while *IL1A*, *CXCL2*, and *IFNG* were more greatly expressed by individuals on the opposite side (Fig. 3D).

### Immune functional assay as a stratification tool for sepsis patients

**Figure 4:**
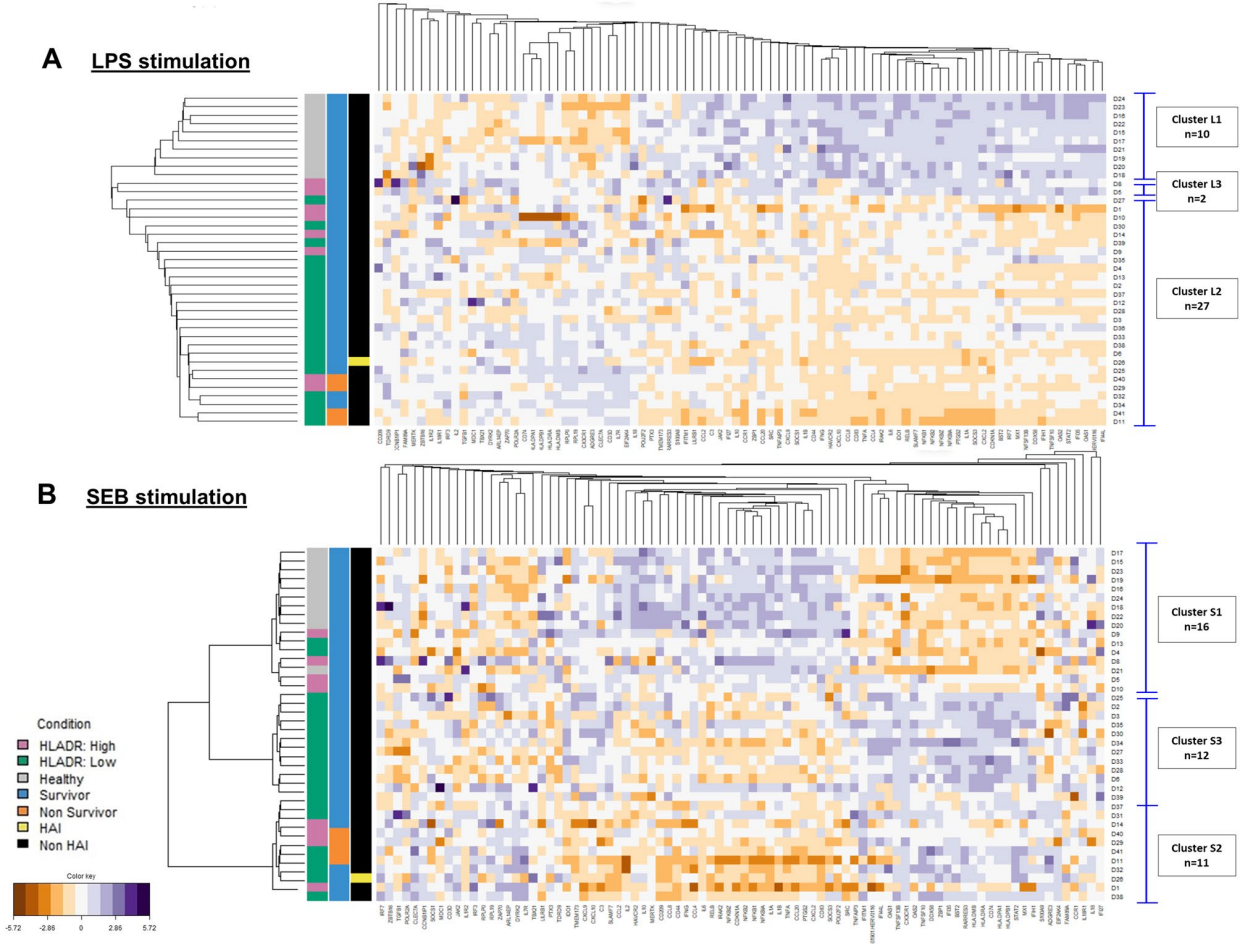
Multivariate clustering analysis upon LPS and SEB stimulation. 10 healthy volunteers and 30 septic shock patients were treated as a whole to discriminate associative gene patterns. (**A**) LPS response revealed 3 clusters of individuals (L1; n = 10, L2; n = 27 and L3; n = 2) using hierarchical method and manhattan distances. (**B**) SEB response revealed 3 clusters in the discovery cohort (S1; n = 16, S2; n = 11 and S3; n = 12) using PAM method with correlation distance. The homemade dendogram is based on the distance between the individuals from the medoid of each cluster found by PAM. Darker purple colours on the heat map indicates higher fold change for upregulated genes (stimulation/control condition), while darker orange colours indicate higher fold change for downregulated genes. 10,000 AB/c was used as a threshold for high and low mHLA-DR. *LPS* lipopolysaccharide, *SEB* staphylococcal enterotoxin B, *HLA-DR* human leukocyte antigen DR, *HAI* hospital-acquired infection.

Taking into consideration the healthy and the septic shock populations, we performed unsupervised clustering with the entire molecular panel in order to identify gene patterns. HV were clustered together after LPS and SEB stimulation, showing high homogeneity in the immune response. Upon LPS stimulation, with the exception of two patients (n = 2, cluster L3), septic shock patients mainly constituted a single cluster (n = 27, cluster L2; Fig. 4A). Upon SEB stimulation 6 septic shock patients clustered with HV (n = 16, cluster S1) and the others were separated into two groups of almost equal number (n = 11, cluster S2 and n = 12, cluster S3; Fig. 4B). The composition of each cluster is presented in Supplementary Table S5.

Bivariate analysis was then performed between clusters and laboratory or clinical parameters. For LPS stimulation, cluster L1 was only composed of HV and cluster L3 only included 2 individuals, precluding statistical analysis for clinical parameters. LPS-induced alterations in septic shock patients were profound and common to all subjects, precluding further separation of patients based on the latter. For SEB stimulation, statistical significance was found for mHLA-DR (*p* value = 0.007) and for TNFα protein secretion post-LPS stimulation (*p* value < 0.001; Table 2). As expected from clustering with HV, the 6 septic shock patients contained in cluster S1 had the highest median values of mHLA-DR (10,938 AB/c, IQR: [9456–14642]) and TNFα protein concentration post-LPS stimulation (3799 pg/mL, IQR: [2067.2–5401.2], values include those for HV) compared to the remaining septic shock patients. Comparing cluster S1 to S2, the only significant difference was the median TNFα protein concentration post-LPS stimulation (*p* value < 0.001); cluster S2 had the lowest median level among the 3 clusters. Comparing cluster S1 to S3, there was a significant difference for both TNFα protein concentration and mHLA-DR (both *p* value < 0.001); among the 3 clusters S3 had an intermediate median level of TNFα protein concentration post-LPS stimulation clusters, while median levels of mHLA-DR were lowest in S3 (supplementary Figure S3). There was a trend towards a shorter ICU length of stay according to the severity of the cluster-based immunotypes (*p* value = 0.585). Septic shock patients in cluster S2 had a longer ICU stay and were more severe than those in other clusters; this cluster included the 4 non-survivors at day 28, representing 36% of this cluster (*p* value = 0.034; Table 2), as well as the only patient who developed a hospital-acquired infection (HAI). Length of ICU stay for septic shock patients in cluster S3 closely followed that of those in cluster S2, while the shorter stay was observed for septic shock patients in S1 who clustered together with HV. Of note, no significant difference was found for hydrocortisone treatment between the assessed profiles. Contrary to LPS, SEB stimulation thus showed a heterogeneous immune response among septic shock patients that allowed to characterise specific immunotypes correlated with disease severity.

**Table 2:** Bivariate analyses between clusters S1, S2, and S3 upon SEB stimulation and clinical and biological parameters.

	Cluster S1 (n = 16)	Cluster S2 (n = 11)	Cluster S3 (n = 12)	*p* value
Status				**< 0.001**
Healthy, n (%)	10 (62.5)	0 (0)	0 (0)	
Patients, n (%)	6 (37.5)	11 (100)	12 (100)	
Comorbidities*^,a^				0.171
No, n (%)	1 (16.7)	6 (54.5)	2 (16.7)	
Yes, n (%)	5 (83.3)	5 (45.5)	10 (83.3)	
Median SOFA* (day 1), [IQR]	7.5 [6.2–8]	8 [6.5–10.5]	8.5 [8–10]	0.574
Median ICU length of stay*, [IQR]	4.5 [4–5.8]	10 [7.5–24]	9 [6.5–12]	0.585
Mortality at day 28*, n (%)	0 (0)	4 (36.4)	0 (0)	**0.034**
Median mHLA-DR* (day 3–4) (Ab/C), [IQR]	10,938 [9456–14642]	7301 [4653–11673]	3839.5 [3444–6250]	**0.007**
Median TNFα secretion post-LPS stimulation (pg/mL), [IQR]	3799 [2067.2–5401.2]	282.7 [122.2–861.8]	700.8 [457.8–913.3]	**< 0.001**

For categorical variables Chi-squared test was used and for numerical variables, *t* test (parametric) or Wilcoxon (non-parametric) was used.

*SOFA* sequential organ failure assessment, *ICU* intensive care unit, *HLA-DR* human leukocyte antigen DR, *TNFα* tumour necrosis factor alpha, *LPS* lipopolysaccharide.

*Parameters measured exclusively for septic shock patients.

^a^Presence of comorbidities was affirmative when at least one of the following comorbidity was present in the patient: chronic pulmonary disease, heart failure, myocardial infarction, ulcer, diabetes, renal failure, or malign solid tumour.

### Validation of the SEB-based IFA clustering in an independent cohort of sepsis patients and HV

#### Description of the validation cohort

From October 2017 to March 2018, 10 new HV and 30 new sepsis patients (with or without shock) were included prospectively according to the Sepsis-3 definition; 67% of the cohort were septic shock patients at inclusion. The main differences with the first cohort of patients was a lower median [IQR] SAPS II score (47 [39–56], *p* value = 0.002), the primary site (*p* value = 0.058) and type of infection (*p* value = 0.068), and a shorter hospital length of stay (28 days [13–48], *p* value = 0.025) despite a greater frequency of HAI (26.7% vs. 3.3%, *p* value = 0.025). Further details on clinical and immunological characteristics of HV and septic patients are described and compared to the first cohort in Supplementary Table S6. Presence and nature of comorbidities are presented in Supplementary Table S7, and number of infections and pathogen responsible for sepsis are presented in Supplementary Table S8.

#### Validation of the SEB-based IFA clustering

To evaluate the robustness of the SEB-based IFA clustering identified in the discovery cohort, identical in vitro stimulation and bioinformatic processes were applied to this new cohort. Comparison of responses between septic patients and HV confirmed the distinct modulation of genes previously observed in the discovery cohort. The genes for which the expression was specifically increased in septic shock patients included in the discovery cohort post-SEB stimulation were similarly dysregulated in the validation cohort (*BST2*, *CD74*, *HLA-DPA1*, *HLA-DR*, *RARRES3*, and *STAT2*; *p* value < 0.05).

**Figure 5:**
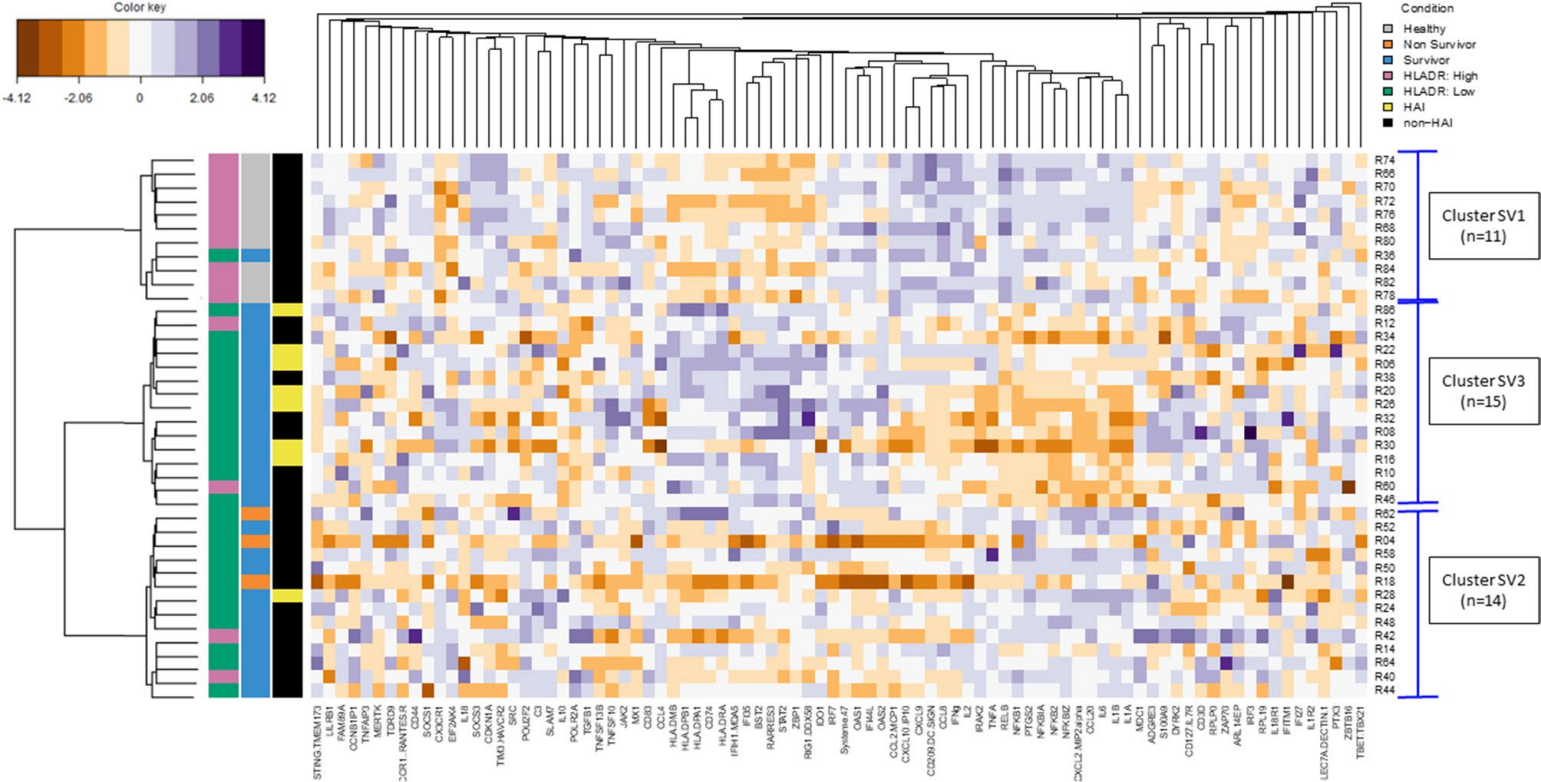
Multivariate clustering analysis upon SEB stimulation in the validation cohort. 10 healthy volunteers and 30 septic patients were treated as a whole to discriminate associative gene patterns. SEB response revealed 3 clusters (SV1; n = 11, SV2; n = 14 and SV3; n = 15) using PAM method with correlation distance. The homemade dendogram is based on the distance between the individuals from the medoid of each cluster found by PAM. Darker purple colours on the heat map indicates higher fold change for upregulated genes (stimulation/control condition), while darker orange colours indicate higher fold change for downregulated genes. 10,000 AB/c was used as a threshold for high and low mHLA-DR. *SEB* staphylococcal enterotoxin B, *HLA-DR* human leukocyte antigen DR, *HAI* hospital-acquired infection.

Cluster building was performed on the validation cohort and 3 clusters were obtained, the composition of each is presented in Supplementary Table S9. The first cluster, SV1, grouped together all the HV and one septic patient (R36). The latter had high TNFα protein concentration post-LPS stimulation (6234 pg/mL), similar to the that of HV (median [IQR]: 4144 pg/mL [3622–4956]), but low levels of mHLA-DR (2975 AB/c) compared to HV (median [IQR]: 28,344 AB/c [21238–31404]). The remaining patients (n = 29) were split into 2 clusters: cluster SV2 (n = 14) included all non-survivors at day 28 (n = 3) who did not develop HAI as well as 1 patient who developed a HAI, and cluster SV3 (n = 15) included 7/8 (87.5%) patients who developed a HAI. Once more, a severity-based grouping was obtained following SEB stimulation (Fig. 5 and Table 3) and a discordance between TNFα post-LPS stimulation and mHLA-DR was again observed (supplementary Figure S4). Of note, no significant difference was found for hydrocortisone treatment between the assessed profiles.

**Table 3:** Bivariate analyses between clusters SV1, SV2, and SV3 upon SEB stimulation and clinical and biological parameters.

	Cluster SV1 (n = 11)	Cluster SV2 (n = 14)	Cluster SV3 (n = 15)	*p* value
Status				**< 0.001**
Healthy, n (%)	10 (91)	0 (0)	0 (0)	
Sepsis patients, n (%)	0 (0)	6 (43)	4 (27)	
Septic shock patients, n (%)	1 (8)	8 (57)	11 (73)	
Comorbidities*^,a^				0.427
No, n (%)	0 (0)	5 (35.7)	3 (20)	
Yes, n (%)	1 (100)	9 (64.3)	12 (80)	
Day 1				
Median SOFA*, [IQR]	8	9 [8–9.8]	9 [4.5–10.5]	0.335
Day 3–4				
Median mHLA-DR (Ab/C), [IQR]	28,272 [18308–30940]	5098 [3544–8223]	4680 [3097–8709]	**< 0.001**
Median TNFα secretion post-LPS stimulation (pg/mL), [IQR]	4176 [3644–5412]	1590 [1289–2442]	719 [474–1090]	**< 0.001**
Outcomes				
Hospital-acquired infections*, n (%)	0 (0)	1 (7.1)	7 (46.7)	**0.035**
Median ICU length of stay*, [IQR]	2	7.5 [5–9.8]	11 [7–16]	**0.050**
Mortality at day 28*, n (%)	0 (0)	3 (21.4)	0 (0)	**0.053**

For categorical variables Chi-squared test was used and for numerical variables, *t* test (parametric) or Wilcoxon (non-parametric) was used.

*SOFA* sequential organ failure assessment, *ICU* intensive care unit, *HLA-DR* human leukocyte antigen DR, *TNFα* tumour necrosis factor alpha, *LPS* lipopolysaccharide.

*Parameters measured exclusively for septic patients.

^a^Presence of comorbidities was affirmative when at least one of the following comorbidity was present in the patient: chronic pulmonary disease, heart failure, myocardial infarction, ulcer, diabetes, renal failure, or malign solid tumour.

## Discussion

In the present study, 2 cohorts of septic patients, recruited according to the Sepsis-3 definition involving dysregulated host response, were analysed using an IFA to evaluate the response capacity of cells, independently of the previous pathogen exposure, and to stratify patients according to their immune alteration-based profiles.

The dysfunctional immune response of septic shock patients identified by the IFA was characterised by fewer differentially expressed genes upon stimulation and therefore fewer modulated canonical pathways. Interestingly, the genes, and consequently the enriched pathways, were differently affected by the two stimuli investigated. For LPS stimulation, there were less than 20% of genes modulated (and thus 95% less modulated pathways) as compared to HV, revealing a profound alteration of the innate arm induced by LPS. This suggests a state of immunoparalysis at day 3–4 after shock onset, as previously described^33,34^. Among the genes expressed differentially between HV and septic shock patients, *NFκB*-related genes allowed discrimination of these groups, highlighting the previously reported down-regulation of this pathway in septic patients^35,36^. Interestingly, genes of the *HLA* family were able to divide the larger group of septic shock patients, indicating that mHLA-DR expression, the only marker used to determine the immune status of sepsis patients^37,38^, could potentially be used to divide the septic population. However, the number of individuals studied herein was too low for statistical analysis. This LPS-based IFA revealed innate immune alterations, common to all sepsis patients, previously described in independent studies, confirming that IFAs are reliable tools that can be successfully used in the septic population.

Conversely, SEB stimulation showed a different picture; a high number of genes and pathways were still able to be induced in septic patients of the discovery and validation cohorts. Activation of HMGB1 or TREM1 pathways, described as up-regulated in sepsis^39,40^, indicated that cells were still able to mount a pro-inflammatory defence after a challenge. We also observed that SEB induced down-regulation of two known anti-inflammatory pathways (PPAR and LXR/RXR) described in sepsis-related contexts^32,41^ and atherosclerosis^42^. It remains to be determined whether the differential response to LPS and SEB stimulation is a consequence of a different altered state that depends on the immune cell population, or whether SEB, because of its superantigen nature triggering innate and adaptive response, exceeds a threshold of activation which LPS is not able to surpass. Nonetheless, the results of the present study indicate that in septic shock patients there is a strong dysregulation of the innate arm induced by LPS compared to the adaptive arm induced by SEB, in accordance with recently published results^4^. Furthermore, heterogeneity of the host immune response in septic shock patients was unmasked by SEB stimulation and this is found in the adaptive arm.

In the discovery cohort, 3 groups of patients characterised by a different level of severity were observed, and validation of these results were obtained using an independent cohort of HV, older than those of the discovery cohort, and a broader and more heterogeneous sepsis cohort which included not only shock patients. The strong correlation obtained with a previous independently published study emphasized the robustness and reproducibility of the overall protocol^43^, and the 42 “sepsis-related host response” genes added to the evaluation panel were found to be informative and gave a more precise picture of the functional capacity of the immune cells in context of sepsis. The clusters S1 and SV1, that could be considered as the “healthier” clusters, included all HV together with a variable number of septic patients (n = 6 and n = 1 in the discovery and validation cohorts, respectively). Additionally, the “healthier” cluster was characterised in both cohorts by the expression of genes that belong to the 44-gene signature identified in immunocompetent individuals^44^ and by up-regulation of genes usually described as being altered in sepsis such as *NFκB*-related genes^36^, *DC-SIGN/CD209*^45^, and *MERTK*^32^. This supports the observation that the septic patients in this cluster had a healthier immune profile than the other patients of the study. The clusters S2 and SV2, that could be considered as the “severe” clusters, included all the non-survivors at day 28 who did not develop any HAI before death (n = 4 in the discovery cohort and n = 3 in the validation cohort). Of note, genes previously described in subgroups of septic patients as being associated with mortality were, herein, found to be modulated specifically in the “severe” cluster in the discovery (*DIRK2*, *ADGRE3*, *CCNB1IP1*^30^, and *IL7R/CD127*^46^) and validation (*MDC1*^30^ and *IFI44L*^47^) cohorts. This could account for an unequal distribution in the initial infectious site as it is reported that the expression of these genes is impacted by the site of the initial infection in sepsis^47^. In accordance, the identified “severe” cluster gathers the expression of genes associated with poor outcome and high mortality described in previous studies^30,46–48^. Interestingly, the SEB-based IFA also identified S3 and SV3 clusters of septic patients who seem to display an intermediate-to-severe phenotype. This “intermediate” cluster included the majority of patients who developed a HAI (n = 7/8) in the validation cohort. The molecular phenotype of this “intermediate” cluster consisted, both in the discovery and validation cohorts, in the up-regulation of the *HLA* family (*HLD-DR*, *HLA-DP*, *HLA-DM*, and *CD74*), usually down-regulated in septic patients^49,50^, and up-regulation of interferon-related genes, a pathway already associated with a lower risk of worsening in a previous study^48^. In addition, the up-regulation of *BST2*, *IFI35*, and *TNFSF13B* that belong to the 44-gene signature reflecting healthy immunity^44^, points to the possibility of a certain degree of immune recovery, and suggests that patients from this cluster may benefit from immunostimulatory therapy. Overall, SEB-based IFA, which identified 3 groups distinguished by severity, allowed to better capture the composite response of septic patients rather than the commonly used mHLA-DR and TNFα post-LPS stimulation markers for immune-monitoring in sepsis^25,37^ or inclusion criteria in clinical trials^10,51^.

There are some limitations and further considerations to this study. The results obtained herein are a reflection of cell activation by the selected stimuli. Nevertheless, we strongly believe that SEB, by connecting both innate and adaptive arms, may be a generic stimulus allowing to evidence the global immune system reactivity. Moreover, the SEB-based IFA should be assessed at different time-points during the course of sepsis, starting at admission, to determine the most appropriate moment to conduct this assay as well as to study the evolution of the patient´s response over time. Likewise, parallel evaluation of the transcriptomic signatures identified at steady state on whole blood with transcriptomic profiling ex vivo post-stimulation from the same patient should be performed. Accordingly, further investigations are required to understand whether there is an added value of performing an IFA in order to personalise care of septic patients. Due to the time-consuming aspect of this test, ways of simplifying and optimising the protocol have been explored to reduce time-to-results. Preliminary results indicate that the stimulation time can be decreased by up to 4 h, while preserving the informative value of the gene panel, and RNA preparation and detection of gene expression by up to 1 h using the automated FilmArray. Although additional validation is required, the possibility of shortening the time required to perform the test and thus to obtain the results within a day is consistent with the requirements of implementation in routine clinical practice, as previously discussed^52^. Moreover, the number of patients included in the present study was limited and therefore the results require further validation on a larger sepsis population.

Heterogeneity in the response of septic patients facing different immune challenges is evidenced herein. The SEB-based IFA, which allowed to capture the diversity of the immune response in a sepsis population, appears to offer an added value allowing an additional stratification of the sepsis population compared to a single static parameter, such as mHLA-DR. The identification of a category of patients with a recovery potential could make this assay a suitable instrument for evaluating the course of sepsis at an individual level. This could represent the starting point to personalise care in sepsis.

## Methods

### Study population

#### Discovery cohort

This clinical study was conducted on septic shock patients admitted to the ICU of the Edouard Herriot hospital (*Hospices Civils de Lyon*, HCL, Lyon, France) and is part of a wider study on ICU-induced immune dysfunctions (NCT02803346)^53^. It was approved by the regional ethics committee (*Comité de Protection des Personnes Sud-Est II*, number 11236), which waived the need for written informed consent owing to the observational nature of the study, with a low risk for patients, and no specific procedure was required other than routine blood sampling. Oral information and non-opposition to inclusion in the study were mandatory, and were systematically obtained before any blood sample was drawn. This was recorded in the patients’ clinical file. This study was also registered at the French ministry of research (*Ministère de lʼEnseignement supérieur, de la Recherche et de lʼInnovation*, DC-2008–509) and with the national data protection commission (*Commission Nationale de l’Informatique et des Libertés*). The study protocol was designed and conducted in accordance with the Declaration of Helsinki^54^ and Good Clinical Practice.

Patients with septic shock were included prospectively. Septic shock was defined according to the Sepsis-3 consensus of the Society of Critical Care Medicine and the European Society of Intensive Care Medicine^2^: vasopressor requirement and serum lactate > 2 mmol/L in the absence of hypovolemia in a patient with suspected or proven infection. The exclusion criteria were age < 18 years and the presence of aplasia or known immunosuppressive disease. At admission, data collected included demographic characteristics (age, sex) and site of primary infection; the initial severity was assessed by the simplified acute physiology score (SAPS II; range 0–163) at admission. Death during ICU stay was collected, and the severity 24 h post-admission was assessed using the sequential organ failure assessment (SOFA) score (range 0–24). Measurement of mHLA-DR and TruCulture stimulation were performed on fresh blood, at day 3–4 after septic shock onset, a time-point of the sepsis course for which the well-accepted marker mHLA-DR is able to discriminate immunosuppressed levels from an immunocompetent status^55^.

Concomitantly, blood samples from healthy volunteers (HV) were obtained from the national blood service (*Etablissement Français du Sang*) and used immediately. According to the EFS standardised procedures for blood donation and to provisions of the articles R.1243–49 and following ones of the French public health code, a written non-opposition to the use of donated blood for research purposes was obtained from HV, as previsouly described^53^. The blood donors’ personal data were anonymised before transfer to our research laboratory. We obtained the favourable notice of the regional ethics committee (*Comité de Protection des Personnes Sud-Est II*) and the acceptance of the French ministry of research (*Ministère de lʼEnseignement supérieur, de la Recherche et de lʼInnovation*, DC-2008–64) for handling and conservation of these samples.

#### Validation cohort

REALISM is a prospective longitudinal, single-centre observational study, conducted in the Anaesthesiology and Intensive Care Department at the Edouard Herriot hospital (HCL, Lyon, France)^56^. Among the REALISM subjects, only HV and septic patients (with and without shock) were included in this study. During screening, only primary septic shock patients were included (vasopressors should have been initiated within the first 48 h after ICU admission). The study protocol was approved by the regional ethics committee (*Comité de Protection des Personnes Sud-Est II*, number 2015–42-2). This clinical study was also registered on clinicaltrials.gov (NCT02638779). Written informed consent was obtained from each healthy donor and patient or his/her relative upon inclusion in this study.

### Laboratory measurements

#### mHLA-DR measurement by flow cytometry

The expression of circulating monocyte HLA-DR was determined at day 3–4 after inclusion in the study on peripheral whole blood collected in EDTA-coated tubes. Anti-HLA-DR/Anti-Monocyte Quantibrite assay (BD Biosciences, San Jose, USA) was used on a Navios flow cytometer (NAVIOS; Beckman-Coulter, Brea, CA, USA) and flow data were analysed using Navios software (Beckman Coulter). Monocytes were first gated out from other cells on the basis of CD14 expression and mHLA‐DR expression was then measured on their surface (mono‐parametric histogram) as median of fluorescence intensity related to the entire monocyte population (as recommended by manufacturer)^57^. These results were then transformed into antibodies bound per cell (AB/c) thanks to calibrated standard curve determined with phycoerythrin (PE)‐beads (BD QuantiBrite™ ‐ PE Beads, Becton Dickinson). Results are expressed as AB/c, AB/c < 15,000 being the threshold for immunocompetence^58^.

### Immune functional assay

#### TruCulture stimulation

Heparinised-whole blood (1 mL) from patients, collected at day 3–4 after inclusion in the study and from HV was distributed into pre-warmed TruCulture tubes (Myriad Rbm, Austin, TX, USA) containing the medium alone (NUL), or the medium with LPS (100 ng/mL, *E.coli* O55:B5), or the medium with SEB (400 ng/mL). The concentration, quality, and activity of the LPS is guaranteed by the manufacturer Myriad Rbm^56^. As previously described, these were then inserted into a dry block incubator and maintained at 37 °C for 24 h. Following incubation, the cellular pellet was resuspended in 2 mL TRI Reagent LS (Sigma-Aldrich, Deisenhofen, Germany), vortexed for 2 min, and rested for 10 min at room temperature before storage at -80 °C^44^; the supernatant was also stored at -80 °C for further analysis.

#### Gene expression analysis

Briefly, after on-column mRNA extraction, expression was evaluated using an 86-gene panel (Supplementary Table S10) using the NanoString technology. Data treatment and normalisation were performed using nSolver analysis software (version 4.0, NanoString technologies; see Supplementary Methods). Finally, the ratios between the stimulation condition (either LPS or SEB) and the control condition (NUL) were calculated in order to compare gene expression changes between septic patients and HV. Results are expressed in counts and fold change induction. One LPS TruCulture tube and one SEB TruCulture tube did not pass the quality control and were not included in the analysis of the discovery cohort, as were two SEB TruCulture tubes in the validation cohort.

The response from HV (discovery cohort) evaluated using the 44-gene signature was compared to that of a previously published healthy cohort after a 24-h stimulation on whole blood in pre-filled TruCulture tubes using the same protocol^44^.

#### Protein detection

TNFα protein from septic patients and HV in TruCulture supernatant was quantified using ELLA nanofluidic system (Biotechne, Minneapolis, MI, USA), according to the manufacturers’ instructions. Results are expressed in pg/ml.

#### Statistical analysis and cluster building

Results are expressed as median and interquartile ranges [IQR] for continuous variables. Parametric data was analysed using ANOVA and non-parametric data was analysed using the Kruskal–Wallis test. Spearman’s rank correlation coefficient was used for correlation analysis. Statistical analyses were conducted using GraphPad Prism software (version 5; GraphPad software, La Jolla, CA, USA) and R (version 3.5.1). A *p* value < 0.05 was considered as significant. The false discovery rate method was used to adjust *p* values in gene analysis. Principal component analysis (PCA) was carried out using Genomics Suite 7 (Partek, St. Louis, MO, USA). Enrichment of biological pathways was analysed using ingenuity pathway analysis (IPA; Qiagen, Redwood City, CA, USA). Cluster analysis is further detailed in Supplementary Methods.

### Poster presentation

29th ECCMID congress, April 2019, Amsterdam, Netherlands. 2019 ESS/IFSS congress, October, Chania, Crete.

## Supplementary information

Supplementary information.

## Data Availability

The datasets generated during the current study are available from the corresponding author upon reasonable request.
