# Supplementary material for: Deciphering heterogeneity of septic shock patients using immune functional assays: a proof of concept study
Source: Sci Rep. 2020 Sep 30;10:16136. doi: 10.1038/s41598-020-73014-2 (PMC7527338; doi:10.1038/s41598-020-73014-2)
Supplement: Supplementary file 1 — Supplementary information. [file 41598_2020_73014_MOESM1_ESM.pdf]

Deciphering heterogeneity of Septic Shock Patients using Immune Functional Assays: a proof of concept study

Chloé Albert Vega[1,2,*], Guy Oriol[1,2], François Bartolo[1,3], Jonathan Lopez[4], Alexandre Pachot[2], Thomas Rimmelé[5,6], Fabienne Venet[5,7], Véronique Leray[6], Guillaume Monneret[5,7], Benjamin Delwarde[6], Karen Brengel-Pesce[1,2], Julien Textoris[2,5,6], François Mallet[1,2,5,#], Sophie Trouillet-Assant[1, 8, #]

[1]Joint Research Unit Hospices Civils de Lyon-bioMérieux, Hospices Civils de Lyon, Centre Hospitalier Lyon Sud, Pierre-Bénite, 69495 Lyon, France.

[2]Open Innovation and Partnerships Department, bioMérieux S.A., France.

[3]Soladis, Lyon, France

[4]Hospices Civils de Lyon - Plateforme de Recherche de Transfert en Oncologie, Centre Hospitalier Lyon Sud, Pierre-Bénite, 69495 Lyon, France.

[5]EA 7426 Pathophysiology of Injury-Induced Immunosuppression, PI3, Claude Bernard Lyon 1 University-bioMérieux-Hospices Civils de Lyon, Hôpital Edouard Herriot, 69437 Lyon, France.

[6]Hospices Civils de Lyon, Hôpital Edouard Herriot, Anesthesia and Critical Care Medicine Department, 69437 Lyon, France.

[7]Hospices Civils de Lyon, Hôpital Edouard Herriot, Immunology Laboratory, 69437 Lyon, France.

[8]Virpath - Université Lyon, CIRI, INSERM U1111, CNRS 5308, ENS, UCBL, Faculté de Médecine Lyon Est, 7 rue Guillaume Paradin, 69372 LYON cedex 08, France.

#Equally contributed to this work

*Corresponding author

SUPPLEMENTARY INFORMATION

SUPPLEMENTARY FIGURES

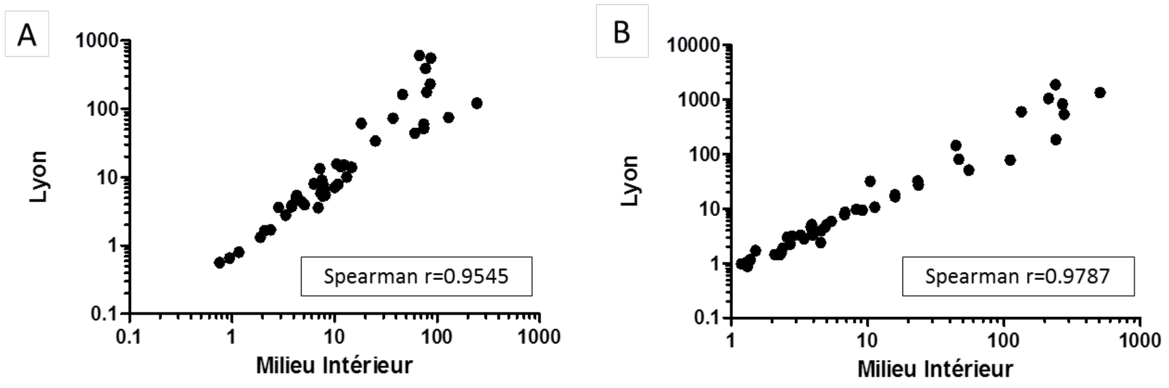

Figure S1. Comparison of response to stimulation between independent studies.

Responses to (**A**) LPS and (**B**) SEB stimulations post-24 hours was evaluated in 10 healthy volunteers representing the control population of the present study referred to as the Lyon study, and in 25 healthy volunteers from the *Milieu Intérieur* study (Urrutia et al. 2016). Comparison of the response to each stimulation between both cohorts was performed on medians of gene expression ratios (stimulation condition / NUL condition) from the 44-gene signature representative of a healthy immunity (Urrutia et al. 2016). Gene expression was quantified using Nanostring nCounter technology and normalised by nSolver software using four housekeeping genes (HPRT1, POLR2A, RPL19 and TBP). The *Milieu Intérieur* data was obtained from Gene Expression Omnibus (GEO) DataSets (GSE85176). A Spearman's correlation coefficient was calculated for each condition and indicated on each graphical representation. LPS: lipopolysaccharide, SEB: staphylococcal enterotoxin B.

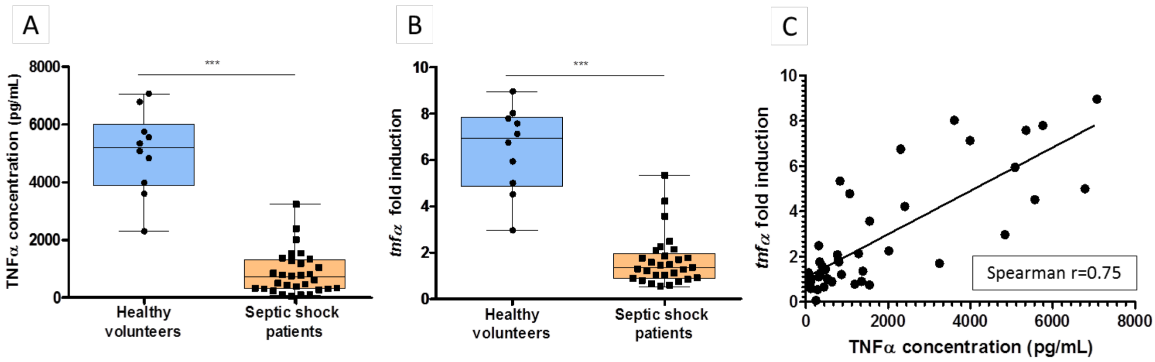

Figure S2. TNFα secreted protein and gene expression between healthy volunteers (HV) and septic shock patients. TNFα response measured in (**A**) protein secretion and (**B**) gene expression to LPS stimulation in 10 HV (blue) and 30 septic shock patients (orange). (**C**) Spearman's correlation coefficient between protein and gene expression for TNFα. ***p<0.001. LPS: lipopolysaccharide.

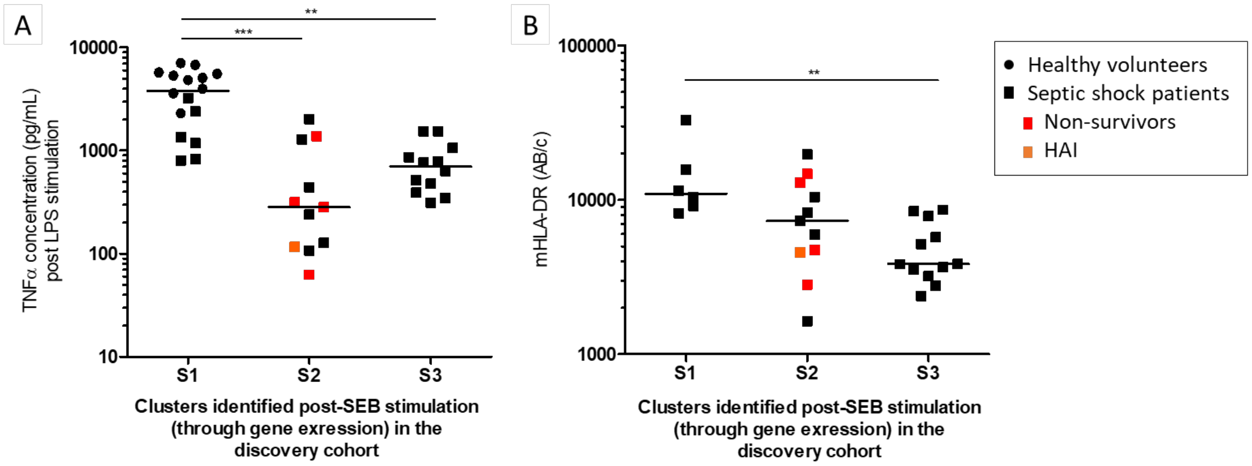

Figure S3. TNFα protein secretion post-LPS stimulation and mHLA-DR distribution in clusters identified upon SEB stimulation (through gene expression) in the discovery cohort. At day 3-4 after septic shock onset, (**A**) TNFα protein secretion was measured *ex vivo* 24 hours post-LPS stimulation in HV (circles) and septic shock patients (squares) and (**B**) mHLA-DR was measured with flow cytometry only in septic shock patients (squares). Mortality is depicted in red and HAI in orange. Clusters post-SEB stimulation were obtained using PAM method with correlation distance. **p<0.001; ***p<0.0001. SEB: staphylococcal enterotoxin B, LPS: lipopolysaccharide, mHLA-DR: monocyte human leukocyte antigen DR, HV: healthy volunteers and HAI: hospital-acquired infection.

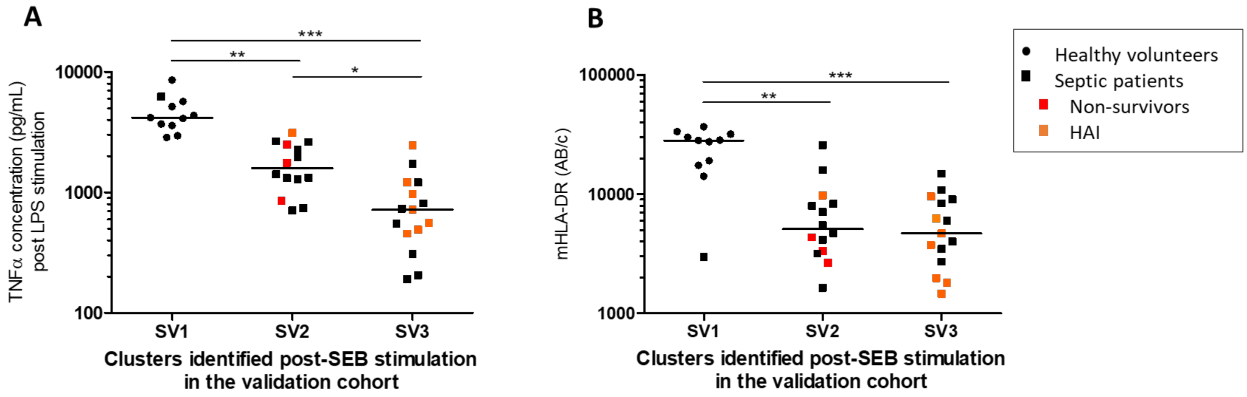

Figure S4. TNFα protein secretion post-LPS stimulation and mHLA-DR distribution in clusters identified upon SEB stimulation (through gene expression) in the validation cohort. At day 3-4 after admission to the study, (**A**) TNFα protein secretion was measured *ex vivo* 24 hours post-LPS stimulation and (**B**) mHLA-DR was measured with flow cytometry in HV (circles) and septic patients (squares). Mortality is depicted in red and HAI in orange. Cluster post-SEB stimulation was obtained using PAM method with correlation distance. **p<0.001; ***p<0.0001. SEB: staphylococcal enterotoxin B, LPS: lipopolysaccharide, mHLA-DR: monocyte human leukocyte antigen DR, HV: healthy volunteers, SP: septic patients and HAI: hospital-acquired infection.

SUPPLEMENTARY METHODS

Experimental pipeline

The pipeline to complete the analysis from blood collection to clustering required 60 hours. In details, 24 hours of blood stimulation, 6 hours for RNA preparation and 27 hours for RNA detection using NanoString protocol and 3 hours of analysis and clustering.

Biological measurements

Molecular detection

For TruCulture cell pellet handling and RNA processing and detection, the protocol was carried out according to the study by Urrutia et al. Cell pellets from TruCulture stimulations kept in TRI Reagent LS (Sigma-Aldrich) were thawed under agitation. Before processing, thawed samples were centrifuged (3.000 g for 5 minutes at 4°C) to pellet cellular debris generated during the Trizol lysis. For extraction, a modified protocol of the NucleoSpin 96 RNA tissue kit (Macherey-Nagel Gmbh&Co. KG, Düren, Germany) was followed using a vacuum system. Briefly, 600 µL of clarified Trizol lysate was transferred to a tube preloaded with 900 µL 100% ethanol. The binding mixture was transferred to a silica column, then washed with buffers MW1 and MW2, and RNA was eluted using 30 µL RNase-free water. NanoString technology was used for mRNA detection of an 86-gene panel (Supplementary Table 1), a hybridisation-based multiplex assay characterised by its amplification-free step; 300 ng of RNA were hybridised to the probes at 67°C for 18 hours using a thermocycler (Biometra, Tprofesssional TRIO, Analytik Jena AG, Jena, Germany). After removal of excessive probes, samples were loaded into the nCounter Prep Station (NanoString Technologies, Seattle, WA, USA) for purification and immobilisation onto the internal surface of a sample cartridge for 2-3 hours. The sample cartridge was then transferred and imaged on the nCounter Digital Analyzer (NanoString Technologies) where colour codes were counted and tabulated for the 86 genes.

Gene expression analysis

Generation of normalised data

Each sample was analysed in a separate multiplexed reaction each including 8 negative probes and 6 serial concentrations of positive control probes. Negative control analysis was performed to determine the background for each sample. Data was imported into nSolver analysis software (version 4.0, NanoString technologies) for quality checking and normalisation of the data. A first step of normalisation using the internal positive controls allowed correction of a potential source of variation associated with the technical platform. To do so, we calculated for all the samples the background level as the median +3 SD across the six negative probe counts. Every sample under the background level was fixed to this value. Next, we calculated for each sample the geometric mean of the positive probe counts. A scaling factor for a sample was a ratio of the geometric mean of the sample and the average across all geometric means. For each sample, we divided all gene counts by the corresponding scaling factor. To normalise for differences in RNA input we used the same method as in the positive control normalisation, except that geometric means were calculated over three housekeeping genes (HPRT1 (NM_000194.1), DECR1 (NM_001359.1), and TBP (NM_001172085.1)). These genes were selected using NormFinder method, an established approach for identification of stable housekeeping genes within and between groups, from the 6 candidate genes included in the custom panel. Results are expressed as fold change inductions.

Cluster building

Data were log10-transformed, centred and scaled. Two distances matrix and a correlation matrix were built on the data and 10 clustering methods were launched ("hierarchical", "k-means", "diana", "fanny", "som", "model", "sota", "pam", "clara", and "agnes"). For each method, k=3 to k=18 clusters were tested. The best clusters methods were selected using 7

indexes combining internal (connectivity, silhouette width, and Dunn index) and stability measures (the average proportion of non-overlap (APN), the average distance (AD), the average distance between means (ADM), and the figure of merit (FOM)). The most stable method for LPS was the hierarchical method using manhattan distances and single-linkage clustering (score index=17), however this method for SEB response had a score index of 541, so the most stable method for SEB was found to be the PAM method using correlation and average distance (score index=31).

SUPPLEMENTARY TABLES

Supplementary Table S1. Presence and nature of patients' comorbidities in the discovery cohort.

Sample	Comorbidities	Cerebro-vascular damage	Pulmonary chronic	Heart failure	Myocardial infarction	Ulcer	Diabetes	Renal damage	Solid tumours malignancy
D1	1	0	1	0	0	1	1	0	0
D2	1	0	0	1	0	0	0	1	0
D3	1	0	0	0	0	0	0	2	0
D4	1	0	0	0	0	1	0	0	0
D5	1	1	0	1	0	0	0	3	0
D6	0	0	0	0	0	0	0	0	0
D8	0	0	0	0	0	0	0	0	0
D9	1	0	0	1	1	0	1	3	0
D10	1	1	0	0	0	0	0	0	1
D11	1	0	0	0	0	0	1	0	0
D12	1	1	0	0	0	0	0	0	1
D13	1	0	1	0	0	0	0	0	1
D14	1	0	0	0	0	1	0	0	2
D25	1	1	0	1	1	0	2	1	1
D26	0	0	0	0	0	0	0	0	0
D27	1	0	0	0	0	0	0	0	1
D28	1	0	1	0	1	0	1	0	1
D29	1	0	1	0	0	0	0	0	0
D30	1	0	1	0	0	0	0	0	0
D31	0	0	0	0	0	0	0	0	0
D32	1	0	0	0	0	0	0	1	0
D33	1	0	0	0	1	0	0	0	1
D34	1	0	0	0	0	0	1	0	0
D35	1	0	0	0	0	0	1	1	2
D36	0	0	0	0	0	0	0	0	0
D37	0	0	0	0	0	0	0	0	0
D38	0	0	0	0	0	0	0	0	0
D39	0	0	0	0	0	0	0	0	0
D40	0	0	0	0	0	0	0	0	0
D41	0	0	0	0	0	0	0	0	0

Supplementary Table S2. Infection number, type, and germ responsible for sepsis in the discovery cohort.

Sample	Infection number	Type	Germ
D1	1	Gram-negative	*Escherichia* coli
D2	1	Gram-negative	*Escherichia* coli
D3	2	Gram-positive	*Enterococcus faecium and Streptococcus spp*
D4	NA	Non-documented infection	
D5	1	Gram-negative	*Proteus mirabilis*
D6	2	Gram-negative and Gram-positive	*Ungroupable Streptococcus* and *Klebsiella pneumoniae*
D8	NA	Non-documented infection	
D9	NA	Non-documented infection	
D10	1	Gram-positive	*Enterococcus faecium*
D11	NA	Non-documented infection	
D12	2	Gram-negative and Gram-positive	*Escherichia* coli and Enterococcus *spp*
D13	NA	Non-documented infection	
D14	2	Gram-positive and fungal	*Enterococcus faecium* and *Candida tropicalis*
D25	NA	Non-documented infection	
D26	1	Gram-positive	*Streptococcus pyogenes (A)*
D27	NA	Non-documented infection	
D28	1	Gram-negative	*Enterobacter cloacae*
D29	2	Gram-positive	*Lactobacillus spp* and *Enterococcus faecalis*
D30	1	Virus	*Influenza*
D31	2	Gram-positive and fungal	*Staphylococcus epidermidis* and *Candida albicans*
D32	2	Gram-negative and Gram-positive	*Klebsiella oxytoxa* and *Enterococcus faecalis*
D33	1	Gram-negative	*Escherichia coli*
D34	1	Gram-negative	*Escherichia coli*
D35	2	Gram-negative and Gram-positive	*Escherichia coli* and *Enterococcus spp*
D36	NA	Non-documented infection	
D37	2	Gram-positive	*Enterococcus faecalis* and *Enterococcus spp*
D38	1	Gram-negative	*Escherichia coli*
D39	NA	Non-documented infection	
D40	NA	Non-documented infection	
D41	2	Gram-positive	*Ungroupable (viridans) Streptococcus*

Supplementary Table S3. **Weight of genes driving the total variance in first (PC1) and second (PC2) component for LPS stimulation in both populations.** Ordered from the highest to the lowest in absolute value.

**LPS stimulation_PC1**	**weight**	**LPS stimulation_PC2**	**weight**
SLAMF7	-0.1554	CD74	0.3081
NFKBIA	-0.1551	HLA-DPA1	0.3030
NFKB1	-0.1505	HLA-DRA	0.2859
IDO1	-0.1501	HLA-DMB	0.2740
IFIH1	-0.1496	HLA-DPB1	0.2623
NFKB2	-0.1495	BST2	0.1889
NFKBIZ	-0.1494	IL18	0.1741
OAS1	-0.1470	ZBP1	0.1714
121601901-HERV0116	-0.1467	RPLP0	0.1639
RELB	-0.1460	RARRES3	0.1563
CXCL2	-0.1455	TBX21	0.1507
IFI44L	-0.1455	STAT2	0.1432
SOCS3	-0.1451	IRF3	0.1328
IL1A	-0.1431	ARL14EP	0.1313
IRF7	-0.1424	IL6	-0.1305
CXCL10/IP10	-0.1422	OAS2	0.1292
SRC	-0.1415	RPL19	0.1290
ADGRE3	0.1413	IFI35	0.1283
TNFA	-0.1411	MX1	0.1252
IFI35	-0.1400	ZAP70	0.1240
CDKN1A	-0.1395	DYRK2	0.1236
CCL4	-0.1387	IL18R1	-0.1213
IRAK2	-0.1384	MDC1	0.1191
TNFSF10	-0.1378	TNFSF13B	0.1186
IFNG	-0.1369	IL1B	-0.1099
STAT2	-0.1351	PTGS2	-0.1068
DDX58/RIG1	-0.1347	TNFSF10	0.1053
IL1B	-0.1337	OAS1	0.1050
HAVCR2/TIM3	-0.1325	S100A9	-0.1038
SOCS1	-0.1319	IL1R2	-0.1020
CCL8	-0.1317	DDX58/RIG1	0.0930
CD83	-0.1317	IL10	-0.0909
CCR1	-0.1316	JAK2	0.0860
TNFSF13B	-0.1308	CD3D	0.0858
OAS2	-0.1296	CCL20	-0.0849
PTGS2	-0.1254	IL1A	-0.0812
CD44	-0.1194	121601901-HERV0116	0.0782
C3	-0.1193	MERTK	0.0780
RPL19	0.1171	IFIH1	0.0747
CXCL9	-0.1170	SOCS3	-0.0717

IL6	-0.1162	IRF7	0.0707
IFI27	-0.1125	CLEC7A/DECTIN1	0.0695
BST2	-0.1121	CDKN1A	-0.0669
JAK2	-0.1094	NFKBIZ	-0.0657
IL10	-0.1081	EIF2AK4	0.0634
CCL2	-0.1078	IFI44L	0.0622
CX3CR1	0.1054	PTX3	-0.0558
MX1	-0.1052	TDRD9	-0.0534
TNFAIP3	-0.1046	IDO1	-0.0520
CCL20	-0.1040	TNFAIP3	-0.0518
EIF2AK4	0.0920	HAVCR2/TIM3	0.0514
IFITM1	-0.0918	CXCL9	0.0502
CLEC7A/DECTIN1	0.0867	NFKBIA	-0.0490
ZBP1	-0.0850	CXCL2	-0.0462
RPLP0	0.0840	CCL8	0.0427
HLA-DPB1	0.0775	CD209/DC-SIGN	0.0425
DYRK2	0.0770	TGFB1	0.0406
LILRB1	-0.0753	IL7R	0.0397
RARRES3	-0.0707	C3	0.0388
TBX21	0.0689	LILRB1	0.0387
POLR2A	-0.0674	NFKB2	-0.0384
PTX3	-0.0660	NFKB1	-0.0382
POU2F2	-0.0643	ADGRE3	0.0355
ARL14EP	0.0630	POLR2A	-0.0344
IL7R	0.0614	ZBTB16	0.0339
IL18	-0.0605	CD44	-0.0334
CD3D	0.0521	CXCL10/IP10	0.0326
S100A9	-0.0469	POU2F2	0.0310
TMEM173/STING	-0.0452	CCL2	0.0307
IL18R1	0.0430	SOCS1	0.0229
HLA-DRA	-0.0420	CX3CR1	0.0225
HLA-DMB	0.0412	IFNG	0.0220
ZAP70	0.0384	RELB	-0.0210
TGFB1	-0.0382	IRAK2	-0.0185
TDRD9	0.0328	TMEM173/STING	-0.0156
IRF3	0.0317	SLAMF7	-0.0151
MDC1	0.0301	IFI27	0.0150
HLA-DPA1	0.0300	CCR1	0.0121
IL1R2	0.0226	IFITM1	-0.0118
CCNB1IP1	0.0200	FAM89A	-0.0091
ZBTB16	0.0143	TNFA	0.0080
IL2	0.0096	IL2	0.0039
MERTK	-0.0089	CCNB1IP1	-0.0036
FAM89A	-0.0071	CCL4	-0.0032
CD209/DC-SIGN	-0.0035	CD83	-0.0013
CD74	-0.0023	SRC	0.0002

Supplementary Table S4. Weight of genes driving the total variance in first (PC1) and second (PC2) component for SEB stimulation in both populations. Ordered from the highest to the lowest in absolute value.

SEB stimulation_PC1	weight	SEB stimulation_PC2	weight
RARRES3	-0.1761	121601901-HERV0116	0.2284
IL1A	0.1721	SLAMF7	0.2111
CXCL2	0.1675	CCL4	0.2038
STAT2	-0.1658	C3	0.1919
IFNG	0.1633	CXCL10/IP10	0.1880
PTGS2	0.1616	IFIH1	0.1831
CCL20	0.1576	IDO1	0.1793
NFKB1	0.1572	CXCL9	0.1783
ZBP1	-0.1552	IFI44L	0.1778
NFKBIA	0.1535	TNFAIP3	0.1770
HLA-DMB	-0.1522	CD44	0.1735
CD209/DC-SIGN	0.1507	POU2F2	0.1683
IL1B	0.1498	OAS2	0.1658
NFKB2	0.1485	IRAK2	0.1607
NFKBIZ	0.1478	CD74	0.1591
BST2	-0.1477	HLA-DRA	0.1538
TNFSF10	-0.1466	ADGRE3	-0.1522
DDX58/RIG1	-0.1463	OAS1	0.1480
CX3CR1	-0.1458	TNFA	0.1440
HLA-DPB1	-0.1434	SRC	0.1431
CDKN1A	0.1426	HLA-DPA1	0.1429
IL6	0.1423	TNFSF13B	0.1336
IFI35	-0.1403	SOCS1	0.1317
CD83	0.1394	IFI35	0.1304
HAVCR2/TIM3	0.1356	MX1	0.1288
HLA-DPA1	-0.1313	HLA-DPB1	0.1202
TNFA	0.1312	CD83	0.1198
HLA-DRA	-0.1312	TMEM173/STING	0.1188
IL2	0.1302	HLA-DMB	0.1171
IRAK2	0.1294	MDC1	0.1148
RELB	0.1283	CDKN1A	0.1086
CD74	-0.1263	FAM89A	0.1002
IL10	0.1259	NFKB1	0.0965
CCL2	0.1258	NFKBIA	0.0964
SOCS3	0.1228	CCR1	0.0955
OAS2	-0.1216	NFKB2	0.0950
MX1	-0.1161	TBX21	0.0948
TNFSF13B	-0.1127	IL1B	0.0947
IFITM1	-0.1120	CCL8	0.0944
IL1R2	0.1073	BST2	0.0906

DYRK2	-0.1049	IL2	0.0852
RPL19	-0.1011	DYRK2	-0.0834
IL7R	-0.0977	CLEC7A/DECTIN1	-0.0763
CCL8	0.0973	S100A9	-0.0758
TDRD9	0.0966	DDX58/RIG1	0.0754
ARL14EP	-0.0954	MERTK	-0.0730
SRC	0.0944	IL1A	0.0729
MERTK	0.0902	IL7R	-0.0721
CD44	0.0891	ZBTB16	-0.0710
IFIH1	-0.0855	IL10	-0.0694
TMEM173/STING	0.0847	ZBP1	0.0674
CCNB1IP1	-0.0842	RARRES3	0.0663
OAS1	-0.0828	NFKBIZ	0.0663
PTX3	0.0822	PTX3	-0.0658
CLEC7A/DECTIN1	-0.0775	RELB	0.0643
IRF3	-0.0760	IRF3	-0.0620
CCL4	0.0749	ARL14EP	0.0617
EIF2AK4	-0.0740	LILRB1	0.0609
POU2F2	0.0737	IL18R1	-0.0604
LILRB1	0.0719	JAK2	-0.0597
POLR2A	0.0665	CCL20	0.0579
ZBTB16	0.0602	TDRD9	-0.0536
ZAP70	-0.0601	IRF7	0.0490
IDO1	-0.0568	POLR2A	-0.0470
CXCL9	0.0560	IFITM1	0.0463
RPLP0	0.0514	IFNG	0.0461
CD3D	-0.0485	CCL2	0.0427
IL18R1	0.0460	SOCS3	0.0424
IFI44L	-0.0430	HAVCR2/TIM3	-0.0405
TBX21	-0.0422	CD3D	0.0400
C3	0.0361	CX3CR1	0.0385
IRF7	-0.0338	TGFB1	0.0379
JAK2	-0.0296	IFI27	0.0353
TGFB1	0.0285	ZAP70	0.0351
SLAMF7	0.0270	STAT2	0.0323
CCR1	0.0247	IL1R2	-0.0321
FAM89A	-0.0202	CD209/DC-SIGN	0.0319
ADGRE3	0.0177	EIF2AK4	0.0214
IFI27	0.0096	IL6	-0.0154
TNFAIP3	0.0083	RPL19	-0.0153
121601901-HERV0116	-0.0077	CXCL2	0.0146
IL18	-0.0056	CCNB1IP1	0.0120
CXCL10/IP10	-0.0047	TNFSF10	-0.0076
MDC1	0.0043	PTGS2	-0.0072
SOCS1	-0.0036	RPLP0	-0.0068
S100A9	0.0010	IL18	0.0019

Supplementary Table S5. Individual composition of the clusters obtained after LPS and SEB stimulation in the discovery cohort.

CLUSTERS	S1	S2	S3
**L1**	D15; D16; D17; D18; D19; D20; D21; D22; D23; D24		
**L2**	D4; D9; D10; D13	D1; D11; D14; D26; D29; D31; D32; D37; D38 ;D40; D41	D2; D3; D6; D12; D25; D27; D28; D30; D33; D34; D35; D39
**L3**	D5; D8		

Individual composition (donors) of the clusters obtained with LPS stimulation (on rows) and with SEB stimulation (on columns) and correspondence between both stimulations.

HV are depicted in blue, septic shock patients in black, non-survivors in red, and HAI in orange. D7 was excluded from the analysis as it did not pass quality control and there was not enough blood volume to stimulate D36 with SEB. D: Donor

Supplementary Table S6. **Clinical and immunological data for healthy donors and sepsis patients in the discovery and validation cohorts**.

	Discovery cohort (n=40)	Validation cohort (n=40)	P-value
**HEALTHY DONORS**			
**Admission data**			
Sex, male, n (%)	5 (50)	5 (50)	1
median age, years [IQR]	54 [51.5-56.5]	73 [72-73.75]	**<0.001**
Immunology			
median mHLA-DR, Ab/C [IQR]	NA	28344 [21238-31404]	NA
median TNFα secretion post-LPS stimulation, pg/mL [IQR]	5214 [4203-5709]	4144 [3622-4956]	0.464
**SEPTIC PATIENTS**			
**Admission data**			
Sex, male, n (%)	21 (70)	21 (70)	1
median age, years [IQR]	66 [59-73]	66 [60-79]	0.487
median BMI, kg/m2 [IQR]	27 [21-34]	25 [22-28]	0.290
median SAPS II [IQR]	59 [47.5-76.5]	47 [39-56]	**0.002**
median SOFA score (day 1) [IQR]	8 [7-10]	9 [7-10]	0.693
Mechanical ventilation, n (%)	22 (73)	20 (67)	0.778
median plasma Lactate level, mM [IQR]	3.2 [2.6-5.2]	2.2 [1.7-2.6]	**<0.001**
Shock, n(%)	30 (100)	20 (67)	**0.001**
median CCI [IQR]	1.5 [0.1-3.3]	2 [0.8-4]	0.688
Comorbidities[a], n (%)			0.778
0	10 (33.3)	8 (26.7)	
≥1	20 (66.7)	22 (73.3)	
Primary site of infection, n (%)			**0.058**
Abdominal	9 (30)	12 (40)	
UTI	6 (20)	2 (6.7)	
SST	4 (13)	2 (6.7)	
Pulmonary	2(6.7)	10 (33.3)	
Others	9 (30)	4 (12.5)	
Type of primary infection, n (%)			0.068
Community acquired	13 (43)	21 (70)	
Hospital acquired	17 (57)	9 (30)	
Documentation of infection, n (%)			**<0.001**

Gram-negative	7 (23.3)	5 (16.7)	
Gram-positive	6 (20)	10 (33.3)	
Virus	1 (3.3)	0	
Fungal	0	4 (13.3)	
Co-infection	6 (20)	5 (16.7)	
Non-documented infection	10 (33.3)	6 (20)	
Hydrocortisone, n (%)	10 (33)	7 (23.3)	0.566
**Day 3-4 data**			
Immunology			
median mHLA-DR, Ab/C [IQR]	7348 [3838-10103]	4693 [3217-8363]	0.187
median TNFα secretion post-LPS stimulation, pg/mL [IQR]	701 [320-1260]	1214 [709-1911]	**0.021**
**Outcomes**			
Vasopressor requirement, n(%)	30 (100)	28 (93)	0.491
median vasopressor duration, days [IQR]	3.5 [2-6.8]	1.9 [1-3.3]	**0.001**
Hemofiltration, n (%)	10 (33)	7 (23)	0.566
Hospital-acquired infection, n (%)	1 (3.3)	8 (26.7)	**0.025**
median ICU length of stay, days [IQR]	8 [4.2-12]	8 [6-13]	0.378
missing data	0	1	
median hospital length of stay, days [IQR]	56 [20-78]	28 [13-48]	**0.025**
missing data	0	3	
Mortality D28, n (%)	4 (13.3)	3 (10)	1

For numerical variables, t-test (parametric) or Wilcoxon (non-parametric) was used and for categorical variables, Chi-squared test was used.
SAPS II was calculated after admission and SOFA score was measured after 24 h of ICU stay.
BMI: body mass index
SAPS II: simplified acute physiology score
SOFA: sequential organ failure assessment
CCI: Charlson comorbidity index
UTI: urinary tract infection
SST: skin and soft tissue
HLA-DR: human leukocyte antigen DR
TNFα: tumour necrosis factor alpha
LPS: lipopolysaccharide
ICU: intensive care unit
[a]: Presence of comorbidities was affirmative when at least one of the following comorbidity was present in the patient: chronic pulmonary disease, heart failure, myocardial infarction, ulcer, diabetes, renal failure, or malign solid tumour.

Supplementary Table S7. Presence and nature of patients' comorbidities in the validation cohort

Sample	Comorbidities	Cerebro-vascular damage	Pulmonary chronic	Heart failure	Myocardial infarction	Ulcer	Diabetes	Renal damage	Solid tumour malignancy
R3/4	2	0	0	1	0	1	0	0	0
R5/6	1	0	1	0	0	0	0	0	0
R7/8	1	0	0	0	0	1	0	0	0
R9/10	0	0	0	0	0	0	0	0	0
R11/12	3	1	0	1	0	0	0	1	0
R13/14	3	0	0	0	1	0	1	1	0
R15/16	3	0	1	0	0	0	1	1	0
R17/18	2	0	0	1	0	0	0	1	0
R19/20	2	0	0	0	0	0	1	1	0
R21/22	2	0	0	0	1	0	0	0	1
R23/24	0	0	0	0	0	0	0	0	0
R25/26	1	0	0	0	0	0	0	0	1
R27/28	2	0	0	1	1	0	0	0	0
R29/30	2	0	0	0	1	1	0	0	0
R31/32	1	0	0	0	0	0	1	0	0
R33/34	0	0	0	0	0	0	0	0	0
R35/36	5	0	1	1	0	1	1	1	0
R37/38	3	1	0	0	1	0	1	0	0
R39/40	0	0	0	0	0	0	0	0	0
R41/42	0	0	0	0	0	0	0	0	0
R43/44	1	0	0	0	0	0	0	0	1
R45/46	0	0	0	0	0	0	0	0	0
R47/48	3	1	0	1	0	0	0	1	0
R49/50	0	0	0	0	0	0	0	0	0
R51/52	2	0	0	0	0	0	1	1	0
R57/58	0	0	0	0	0	0	0	0	0
R59/60	2	0	0	0	0	0	0	1	1
R61/62	1	0	1	0	0	0	0	0	0
R63/64	1	0	1	0	0	0	0	0	0
R85/86	1	0	1	0	0	0	0	0	0

Supplementary Table S8. Infection number, type, and germs responsible for sepsis in the validation cohort

Sample	Infection number	Type	Germ
R3/4	2	Fungal	*Candida krusei* and *Candida tropicalis*
R5/6	1	Fungal	*Aspergillus fumigatus*
R7/8	3	Gram-negative and fungal	*Enterobacter cloacae, Escherichia coli* and *Candida albicans*
R9/10	2	Gram-positive and other bacteria	*Streptococcus spp* and *Actinomyces spp*
R11/12	2	Gram-negative	*Providencia* and *Escherichia coli*
R13/14	1	Gram-positive	*Staphylococcus aureus*
R15/16	1	Gram-positive	*Staphylococcus aureus*
R17/18	1	Gram-negative	*Klebsiella oxytoxa*
R19/20	NA	Non-documented infection	
R21/22	0	Non-documented infection	
R23/24	1	Gram-negative	*Klebsiella pneumoniae*
R25/26	1	Gram-negative	*Escherichia coli*
R27/28	NA	Non-documented infection	
R29/30	2	Fungal	*Candida albicans* and *Candida glabrata*
R31/32	0	Non-documented infection	
R33/34	2	Gram-positive	*Streptococcus pyogenes (A)* and *Staphylococcus aureus*
R35/36	1	Gram-positive	*Coagulase-negative Staphylococcus spp*
R37/38	3	Gram-negative and Gram-positive	*Escherichia coli, Klebsiella pneumoniae* and *Coagulase-negative Staphylococcus spp*
R39/40	1	Gram-positive	*Gram-positive Bacilli*
R41/42	2	Gram-positive and fungal	*Candida albicans* and *Streptococcus spp*
R43/44	1	Gram-negative	*Klebsiella pneumoniae*
R45/46	1	Fungal	*Candida albicans*
R47/48	NA	Non-documented infection	
R49/50	1	Gram-positive	*Streptococcus pneumoniae*
R51/52	1	Gram-positive	*Enterococcus faecalis*
R57/58	1	Gram-positive	*Staphylococcus aureus*
R59/60	0	Non-documented infection	
R61/62	2	Gram-positive	*Streptococcus spp* and *Staphylococcus epidermidis*
R63/64	1	Gram-positive	*Staphylococcus aureus*
R85/86	2	Gram-negative and Gram-positive	*Streptococcus pneumoniae* and *Moraxella spp*

Supplementary Table S9. Individual composition of the clusters obtained after SEB stimulation in the validation cohort.

Cluster SV1 (n=11)	Cluster SV2 (n=14)	Cluster SV3 (n=17)
R36; R65; R67; R69; R71; R73; R75; R77; R79; R81; R83	R4; R14; R18; R24; R28; R40; R42; R44; R48; R50; R52; R58; R62; R64	R6; R8; R10; R12; R16; R20; R22; R26; R30; R32; R34; R38; R46; R60; R86

HV are depicted in blue, septic patients in black, non-survivors in red, and HAI in orange. R: Donor

Supplementary Table S10. NanoString target genes and accession numbers

Target genes	Accession number	References
ADGRE3	NM_032571.2	Davenport *et al*. *Lancet Respir Med.* 2016
ARL14EP	NM_152316.1	Davenport *et al*. *Lancet Respir Med.* 2016
BST2	NM_004335.2	Urrutia *et al. Cell Rep*. 2016
C3	NM_000064.2	Urrutia *et al. Cell Rep*. 2016
CCL2	NM_002982.3	Urrutia *et al. Cell Rep*. 2016
CCL20	NM_004591.1	Urrutia *et al. Cell Rep*. 2016
CCL4	NM_002984.2	Urrutia *et al. Cell Rep*. 2016
CCL8	NM_005623.2	Urrutia *et al. Cell Rep*. 2016
CCNB1IP1	NM_182849.2	Davenport *et al*. *Lancet Respir Med.* 2016
CCR1	NM_001295.2	Urrutia *et al. Cell Rep*. 2016
CD209/DC-SIGN	NM_021155.2	Pilling *et al. BMC Immunol*. 2017
CD3D	NM_000732.4	Tawfik *et al.* bioRxiv 2019
CD44	NM_001001392.1	Urrutia *et al. Cell Rep*. 2016
CD74	NM_001025159.1	Cazalis *et al*. *Crit Care.* 2013
CD83	NM_004233.3	Urrutia *et al. Cell Rep*. 2016
CDKN1A	NM_000389.2	Urrutia *et al. Cell Rep*. 2016
CLEC7A/DECTIN1	NM_197954.2	Mommert *et al. BMC Genomics.* 2018
CX3CR1	NM_001337.3	Friggeri *et al*. *Crit Care.* 2016
CXCL10/IP10	NM_001565.1	Urrutia *et al. Cell Rep*. 2016
CXCL2/MIP2A	NM_002089.3	Urrutia *et al. Cell Rep*. 2016
CXCL9	NM_002416.1	Urrutia *et al. Cell Rep*. 2016
DECR1	NM_001359.1	Housekeeping gene
DDX58/RIG1	NM_014314.3	This study
DYRK2	NM_003583.3	Davenport *et al*. *Lancet Respir Med.* 2016
EIF2AK4	NM_001013703.2	Mommert *et al. BMC Genomics.* 2018
FAM89A	NM_198552.2	Herberg *et al*. *JAMA.* 2016
HAVCR2/TIM3	NM_032782.3	Gossez *et al. Sci Rep*. 2018
HLA-DMB	NM_002118.3	Urrutia *et al. Cell Rep*. 2016
HLA-DPA1	NM_033554.2	Urrutia *et al. Cell Rep*. 2016
HLA-DPB1	NM_002121.4	Urrutia *et al. Cell Rep*. 2016
HLA-DRA	NM_019111.3	Urrutia *et al. Cell Rep*. 2016
HPRT1	NM_000194.1	Housekeeping gene
IDO1	NM_002164.3	Urrutia *et al. Cell Rep*. 2016
IFI27	NM_005532.3	This study
IFI35	NM_005533.3	Urrutia *et al. Cell Rep*. 2016
IFI44L	NM_006820.2	Herberg *et al*. *JAMA.* 2016
IFIH1	NM_022168.2	Urrutia *et al. Cell Rep*. 2016
IFITM1	NM_003641.3	Urrutia *et al. Cell Rep*. 2016
IFNG	NM_000619.2	Mommert *et al. BMC Genomics.* 2018
IL10	NM_000572.2	Tawfik *et al.* bioRxiv 2019
IL18	NM_001562.2	Tawfik *et al.* bioRxiv 2019

IL18R1	NM_003855.2	Mommert *et al. Crit Care.* 2020
IL1A	NM_000575.3	Urrutia *et al. Cell Rep.* 2016
IL1B	NM_000576.2	Urrutia *et al. Cell Rep.* 2016
IL1R2	NM_004633.3	Mommert *et al. Crit Care.* 2020
IL2	NM_000586.2	This study
IL6	NM_000600.1	Urrutia *et al. Cell Rep.* 2016
IL7R	NM_002185.2	Mouillaux *et al. Crit Care.* 2019
IRAK2	NM_001570.3	Urrutia *et al. Cell Rep.* 2016
IRF3	NM_001571.5	This study
IRF7	NM_001572.3	Urrutia *et al. Cell Rep.* 2016
JAK2	NM_004972.2	Urrutia *et al. Cell Rep.* 2016
LILRB1	NM_001081637.1	Urrutia *et al. Cell Rep.* 2016
MDC1	NM_014641.2	Davenport *et al. Lancet Respir Med.* 2016
MERTK	NM_006343.2	Mommert *et al. BMC Genomics.* 2018
MX1	NM_002462.2	Urrutia *et al. Cell Rep.* 2016
NFKB1	NM_003998.2	Urrutia *et al. Cell Rep.* 2016
NFKB2	NM_002502.2	Urrutia *et al. Cell Rep.* 2016
NFKBIA	NM_020529.1	Urrutia *et al. Cell Rep.* 2016
NFKBIZ	NM_001005474.1	Urrutia *et al. Cell Rep.* 2016
OAS1	NM_001032409.1	Mommert *et al. BMC Genomics.* 2018
OAS2	NM_016817.2	Mommert *et al. BMC Genomics.* 2018
POLR2A	NM_000937.2	Housekeeping gene
POU2F2	NM_002698.2	Urrutia *et al. Cell Rep.* 2016
PTGS2	NM_000963.1	Mommert *et al. BMC Genomics.* 2018
PTX3	NM_002852.3	Albert Vega *et al. Front Immunol.* 2018
RARRES3	NM_004585.3	Urrutia *et al. Cell Rep.* 2016
RELB	NM_006509.2	Urrutia *et al. Cell Rep.* 2016
RPL19	NM_000981.3	Housekeeping gene
RPLP0	NM_001002.3	Housekeeping gene
S100A9	NM_002965.2	Tawfik *et al.* bioRxiv 2019
SLAMF7	NM_021181.3	Urrutia *et al. Cell Rep.* 2016
SOCS1	NM_003745.1	Urrutia *et al. Cell Rep.* 2016
SOCS3	NM_003955.3	Urrutia *et al. Cell Rep.* 2016
SRC	NM_005417.3	Urrutia *et al. Cell Rep.* 2016
STAT2	NM_005419.2	Urrutia *et al. Cell Rep.* 2016
TBX21	NM_013351.1	This study
TBP	NM_001172085.1	Housekeeping gene
TDRD9	NM_153046.2	Davenport *et al. Lancet Respir Med.* 2016
TGFB1	NM_000660.3	Tawfik *et al.* bioRxiv 2019
TMEM173/STING	NM_198282.1	This study
TNFA	NM_000594.2	Tawfik *et al.* bioRxiv 2019
TNFAIP3	NM_006290.2	Urrutia *et al. Cell Rep.* 2016

TNFSF10	NM_003810.2	Urrutia *et al. Cell Rep*. 2016
TNFSF13B	NM_006573.4	Urrutia *et al. Cell Rep*. 2016
ZAP70	NM_001079.3	Davenport *et al*. *Lancet Respir Med.* 2016
ZBP1	NM_001160419.2	Davenport *et al*. *Lancet Respir Med.* 2016
ZBTB16	NM_006006.4	Godini et al. PLoS One. 2018
121601901-HERV0116	chr12:112972627-112975754	Mommert *et al*. *BMC Genomics.* 2018
